# Fat and Fat-Free Mass of Preterm and Term Infants from Birth to Six Months: A Review of Current Evidence

**DOI:** 10.3390/nu12020288

**Published:** 2020-01-21

**Authors:** Constanze Hamatschek, Efrah I. Yousuf, Lea Sophie Möllers, Hon Yiu So, Katherine M. Morrison, Christoph Fusch, Niels Rochow

**Affiliations:** 1 Department of Pediatrics, Paracelsus Medical University, General Hospital, 90471 Nuremberg, Germany; constanzehamatschek@web.de (C.H.); lmoellers123@gmx.com (L.S.M.); Christoph.Fusch@klinikum-nuernberg.de (C.F.); 2 Department of Pediatrics, McMaster University, Hamilton, ON L8S 4L8, Canada; yousufei@mcmaster.ca (E.I.Y.); morriso@mcmaster.ca (K.M.M.); 3 Department of Statistics and Actuarial Science, University of Waterloo, Waterloo, ON L8S 4L8, Canada; honyius@yahoo.com.hk; 4 Department of Pediatrics, University Hospital, 18057 Rostock, Germany

**Keywords:** body composition, lean mass, neonate, nutrition, growth, percentile, trajectory

## Abstract

To optimize infant nutrition, the nature of weight gain must be analyzed. This study aims to review publications and develop growth charts for fat and fat-free mass for preterm and term infants. Body composition data measured by air displacement plethysmography (ADP) and dual energy X-ray absorptiometry (DXA) in preterm and term infants until six months corrected age were abstracted from publications (31 December 1990 to 30 April 2019). Age-specific percentiles were calculated. ADP measurements were used in 110 studies (2855 preterm and 22,410 term infants), and DXA was used in 28 studies (1147 preterm and 3542 term infants). At term age, preterm infants had higher percent-fat than term-born infants (16% vs. 11%, *p* < 0.001). At 52 weeks postmenstrual age (PMA), both reached similar percent-fat (24% vs. 25%). In contrast, at term age, preterm infants had less fat-free mass (2500 g vs. 2900 g) by 400 g. This difference decreased to 250 g by 52 weeks, and to 100 g at 60 weeks PMA (5000 g vs. 5100 g). DXA fat-free mass data were comparable with ADP. However, median percent-fat was up to 5% higher with DXA measurements compared with ADP with PMA > 50 weeks. There are methodological differences between ADP and DXA measures for infants with higher fat mass. The cause of higher fat mass in preterm infants at term age needs further investigation.

## 1. Introduction

Modern neonatology has achieved high survival rates in neonatal intensive care units, allowing clinicians to focus on optimizing infant nutrition and growth. A wide range of discharge weights have been reported in very preterm infants at different hospitals [1,2]. Some centers report, that at term-corrected age, preterm infants could have reached weights that match their term-born counterparts. However, the quality of weight gain in these infants remains unclear. Too little is known about the relative contributions of fat mass and fat-free mass and this needs to be explored further [3,4,5].

There is an emerging body of evidence to suggest that fat mass and fat-free mass are related to long-term outcomes. Ramel et al. showed that in-hospital fat-free mass gain is associated with better neurological and motor outcomes at one year corrected age in very low birthweight infants [6], while Paviotti et al. demonstrated that both fat mass and fat-free mass are related to cerebellar volume at term [7]. Further, consistent with the developmental origin of health and disease (DOHaD) concept, early life body composition is correlated with cardiovascular and metabolic diseases in adulthood [8,9]. This suggests that measuring fat mass and fat-free mass could provide valuable information to clinicians for the optimization of infant nutritional management and growth, and the achievement of desired, long-term outcomes in preterm infants. 

Available techniques for measuring body composition include skinfold thickness, bio-impedance analysis, stable isotope techniques, MRI, dual-energy X-ray absorptiometry (DXA), and air-displacement plethysmography (ADP). Current methods of choice are ADP and DXA, which have proven to be non-invasive, reliable, and accurate techniques for the measurement of (pre-)term infant body composition [10,11]. 

In recent years, several studies have provided data on nutritional intake and body composition for preterm and term infants (Supplementary Table S1 and Table S2). The aim of this review is to compare and analyze the fat and fat-free mass accretion of preterm and term-born infants from recent publications, and to describe age-specific ranges of body composition measurements.

## 2. Materials and Methods 

This study reviewed recent publications that included ADP or DXA measurements of body composition for preterm and term infants until 6 months corrected age. Following the PRISMA (Prevention and Recovery Information System for Monitoring and Analysis) guidelines [12], a search of the PubMed and Web of Science databases was completed using the search terms “body composition” AND “infants”. The literature search was restricted to “humans” and publications published from 1 January 1990 to 30 April 2019. Only publications presenting body composition data measured with ADP or DXA were included in this review. Additionally, included studies required a minimum cohort sample size of 10 infants. In cases where either fat mass or fat-free mass were missing while weight was available, the weight and the available body composition measure were then used to calculate the missing value. Publications without information about postmenstrual age (PMA) or week of life at the time of the body composition measurement were excluded. Only primary publications were included, and no language restrictions were imposed.

The review process was performed using a stepwise approach. First, following retrieval of the publications identified in the literature search, titles and abstracts were screened against the eligibility criteria to select publications for full-text screening. Abstracts were excluded during this stage if infant body composition had not been measured using ADP or DXA or if body composition measurements were only available beyond 6 months corrected age. Following the title and abstract screening, publications underwent a full-text assessment and if they were confirmed to meet the eligibility criteria, available body composition data obtained with ADP or DXA from birth to 6 months corrected age were abstracted. 

Data abstraction was performed by two authors (C.H., E.Y.) using a specially designed Microsoft Excel^®^ spreadsheet, which collected data on the study design, gestational age at birth, birth weight, birth length, birth head circumference, method of body composition measurement, body composition (percent fat mass, fat mass, fat-free mass), chronological age, postmenstrual age, weight, length, and head circumference, country, year(s) of measurement, and sample size. Further clinical data were abstracted on smoking, feeding status, small for gestational age (SGA), appropriate for gestational age (AGA), and large for gestational age (LGA) status, maternal diabetes, and obesity in order to group studies into those that met the criteria for the World Health Organization (WHO) Multicentre Growth Reference Study (MGRS) and those that did not meet the WHO MGRS criteria. The WHO MGRS developed the WHO growth standards, which comprise the infant growth curves routinely used as anthropometric references in clinical care, and according to the study’s eligibility criteria, there had to be no health constraints to growth and mothers had to be willing to consent to exclusively or predominantly breastfeed their child for at least four months. Exclusion criteria were: maternal smoking during pregnancy or lactation, multiple birth, and preterm birth or post-term birth (gestational age at birth less than 37 weeks or beyond 41 + 6/7 weeks [13].

The statistical analysis was conducted using R statistics [14]. The abstracted body composition data were treated as single time points. The distribution of body composition measurements for gestational age were described using the R package GAMLSS (generalized additive model for location scale and shape) [15]. 

## 3. Results

**Figure 1:**
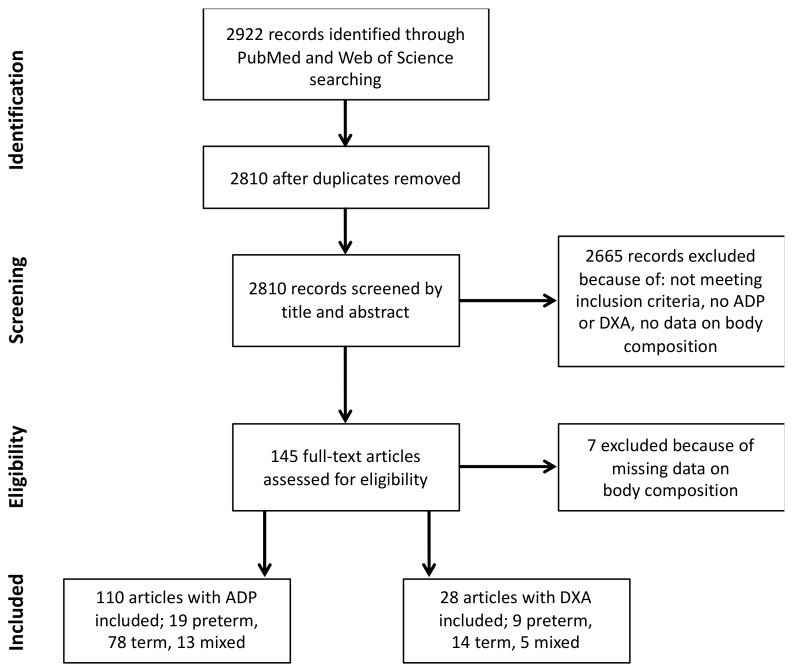
Flow chart of the review process (ADP—air displacement plethysmography, DXA—dual X-ray energy absorptiometry).

The process of this review is illustrated in Figure 1. After the removal of duplicates, 2810 articles were available for review. These articles were screened against the inclusion criteria using the title and abstract. A full-text review was then performed on 146 articles. In total, 138 articles were included in this review, of which 110 articles presented body composition data measured with ADP and 28 articles presented DXA measurements. 

### 3.1. ADP Study Cohorts

Of the 110 articles that provided ADP measures of infant body composition, 19 provided data on preterm infants, 78 on term infants and 13 presented data for both preterm and term infants. Two of the 32 studies exploring preterm body composition presented data on extremely preterm infants (gestational age <28 weeks) and only 21 studies overall provided sex-specific body composition data. Additionally, only five of the included studies met the inclusion criteria for healthy newborns used by the WHO MGRS. In total, these studies reporting ADP compositional outcomes included measurements of 25,265 infants, of which 2855 were born preterm and 22,410 were born full-term. Most of the studies presented data for single time points, with the majority of infants undergoing measurements at term age (between 37 to 41+6/7 weeks) (Table 1). A detailed overview of included studies and health information are presented in the supplemental material (Supplementary Tables S1 and S2).

### 3.2. Percent-Fat Mass, Fat Mass and Fat-Free Mass of Studies Used ADP

**Figure 2:**
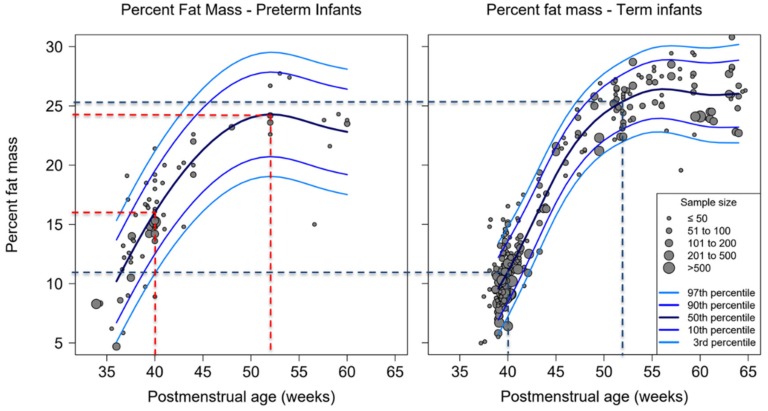
Percentiles of percent-fat mass by postmenstrual age for preterm and full-term infants measured with air-displacement plethysmography (ADP), dots representing average body composition presented by study cohorts, dotted lines indicate percent-fat mass at 40 and 52 weeks postmenstrual age (black—term infants, red—preterm infants). Total number of studies: 110; 19 preterm, 78 term and 13 mixed.

At term preterm infants on average had a higher percent-fat mass than term-born infants at the same age (16% vs. 11%, *p* < 0.001). At 52 weeks PMA, percent-fat mass was comparable between cohorts, with preterm infants reaching 24% and term infants reaching 25%. (Figure 2, Supplementary Table S2). Additionally, their trajectories had similar slopes, although the percent-fat mass trajectory for preterm infants reached approximately 24% at 50 weeks PMA, whereas the curve for term infants reached a plateau with 26% at 54 weeks PMA. (Figure 2 and Supplementary Figures S1 and S3).

**Figure 3:**
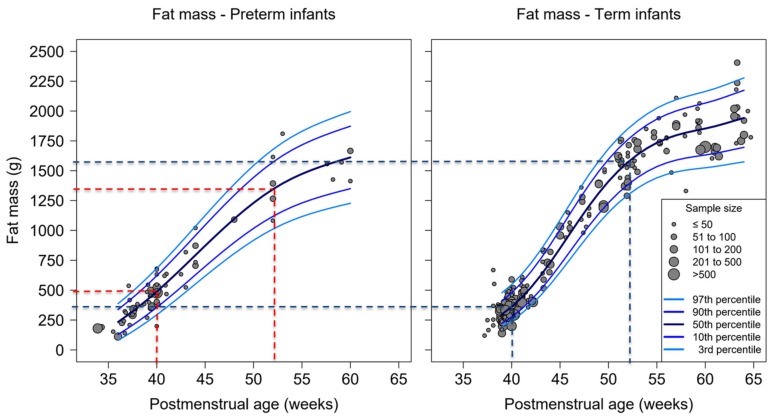
Percentiles of fat mass by postmenstrual age for preterm and full-term infants measured with air-displacement plethysmography (ADP), dots representing average body composition presented by study cohorts, dotted lines indicate percent-fat mass at 40 and 52 weeks postmenstrual age (black—term infants, red—preterm infants). Total number of studies: 110; 19 preterm, 78 term and 13 mixed.

Regarding absolute fat mass at 40 weeks PMA, preterm infants had more fat mass than term-born infants (490 g vs. 360 g). However, at increasing PMAs, the absolute fat mass of term-born infants caught-up with that of preterm infants. The fat mass at 52 weeks of age was 1350 g for preterm infants and 1570 g for term infants. The trajectory of term infants therefore had a steeper course than the one of preterm infants, as shown in Figure 3 and Supplementary Figures S1 and S3.

**Figure 4:**
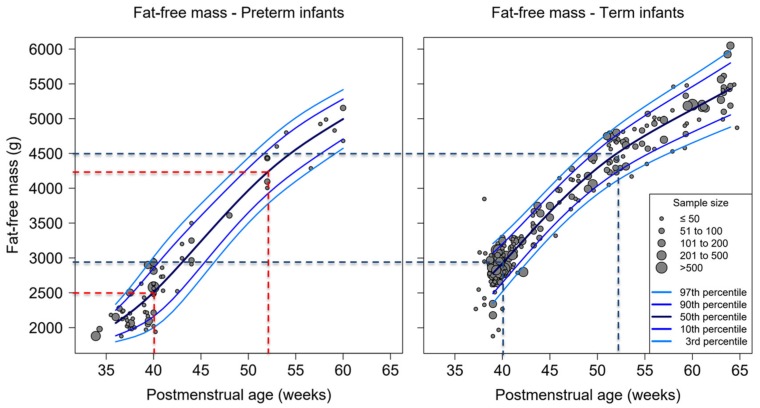
Percentiles of fat-free mass by postmenstrual age for preterm and full-term infants measured with air-displacement plethysmography (ADP), dots representing average body composition presented by study cohorts, dotted lines indicate fat-free mass at 40 and 52 weeks postmenstrual age (black—term infants, red—preterm infants). Total number of studies: 110; 19 preterm, 78 term and 13 mixed.

Fat-free mass at 40 weeks PMA was lower in preterm infants compared with term infants (2500 g vs. 2900 g). However, preterm infants demonstrated catch-up in their fat-free mass with increasing PMA. The difference in average fat-free mass between term-born and preterm infants decreased from 400 g at term age to 250 g at 52 weeks PMA (preterm: 4200 g vs. term-born: 4450 g). At 60 weeks PMA, fat-free mass of preterm infants reached levels of less than 100 g below the average value of term-born infants (5050 g vs. 5130 g). Hence, both curves for fat-free mass converge with increasing PMA until at least 60 weeks PMA (Figure 4 and Supplementary Figures S1 and S3).

At PMA greater than 45 weeks, term infants from studies that met the WHO MGRS inclusion criteria had mean percent-fat mass and fat mass values that were higher than the mean values of term infants from the studies that did not meet the WHO MGRS criteria (Supplementary Figure S3). Fat-free mass, weight, length and head circumference did not differ between these studies (Supplementary Figure S3).

### 3.3. Weight, Length and Head Circumference of Studies Used ADP

As shown in Supplementary Figure S2, preterm infants were lighter than term infants across time-points. This was observed at term corrected age when comparing the average weight of preterm infants to term-born infants (3000 g vs. 3300 g) and at 52 weeks PMA (5400 g vs. 5980 g). By 60 weeks PMA, preterm infants were still around 400 g lighter than term infants (6630 g vs. 7000 g).

Data for length and head circumference were not available for all publications. However, the available data of this review showed that, in parallel with body weight, preterm infants remained shorter than their term-born counterparts. At term corrected age, preterm infants were 2.4 cm shorter than term-born infants (47.9 cm vs. 50.3 cm) and at 52 weeks PMA they were 2.5 cm shorter (58.0 cm vs. 60.5 cm). In contrast to the other parameters, there is little difference in head circumference between term and preterm infants. At 40 weeks PMA, preterm infants had a head circumference that was only 0.5 cm smaller than that of term infants. At 52 weeks PMA, their values were almost the same (40 cm vs. 40.2 cm).

### 3.4. Percent-Fat Mass, Fat Mass and Fat-Free Mass of Studies Used DXA

Body composition data obtained from DXA was available for 28 studies, of which nine presented data on preterm infants, 14 presented data on term infants, and five provided data on both. In total, these papers measured the body composition of 4689 infants, including 1147 preterm and 3542 term-born infants. Detailed information on these studies is provided in the supplemental material (Supplementary Table S5, Supplementary Figure S4). The body composition data measured by DXA show similar trends compared with ADP. However, measurements of percent-fat mass were different between DXA and ADP. At 50 weeks PMA, DXA-measured percent fat mass values were greater by 3% compared to values measured by ADP, reaching a maximum difference of 5% at 65 weeks PMA (Supplementary Figure S5). This was also true for measurements of absolute fat mass, as measurements were higher when measured with DXA, reaching a maximal difference of 500 g at 65 weeks PMA. However, DXA and ADP showed similar values when measuring fat-free mass.

## 4. Discussion

In this study, we compiled a review of published body composition data on preterm and term infants for the construction of growth charts. Our results show that at 40 weeks PMA, preterm infants had a higher percent and absolute fat mass, but lower fat-free mass compared to term infants. At 52 weeks PMA, percent and absolute fat mass were inverted so that term infants had higher values than preterm infants. Furthermore, preterm infants showed a catch-up growth in fat-free mass that almost matched those of term-born infants by 60 weeks PMA.

This review confirms that the normal postnatal development involves a rapid increase in percent-fat mass after birth. Higher percent-fat mass and lower fat-free mass accretion in preterm infants at term age compared to full term infants is consistent with the results of two previous studies [16]. Deficits in fat-free mass among preterm infants appear largely responsible for observed weight differences between groups. The weight difference of 300 g does not accurately reflect the difference in fat-free mass (400 g) at term, because preterm infants have on average 100 g more fat mass compared to term-born infants. This masks the fat-free mass deficit in preterm infants, which is important because fat-free mass is correlated with neurological outcomes, including cerebellar volume [6,7]. This finding is supported by N. Al-Theyab et al. who stated that fat accumulation may be triggered by birth or associated events. They further pointed out the importance of considering this rapid fat accretion, because an assessment of growth based on weight alone would underestimate the deficit in fat-free mass [17]. Therefore, it can be supposed that the use of body composition measurements could be very beneficial for the development of nutritional strategies during infant hospitalization in neonatal intensive care units, and thereby improve fat-free mass at discharge. Body composition measurements at discharge could also guide post-discharge nutritional management, which would allow preterm infants to catch up with the fat-free mass of term-born infants [1].

In this review, full term infants showed more rapid fat mass accrual from term (birth) to 60 weeks PMA compared to preterm infants and had higher average fat mass values and slightly higher percent-fat values. Breast fed infants from studies which matched the WHO MGRS criteria tended to have even higher percent-fat and fat mass compared to the infants from other studies. This finding supports the hypothesis that fat mass accrual may be an adaptive response to the ex-utero environment which may contribute to the higher fat mass and percent-fat in preterm infants at term age, while full term infants had higher levels at 52 weeks PMA. This highlights the differences in growth rates post-term age between preterm and term born infants.

There are several limitations to this review. Firstly, most of the articles included in this review were observational studies. Only a few studies differentiated between male and female infants, and only five cohorts met the inclusion criteria used by the WHO MGRS [13,18,19,20,21,22]. Overall, there was great heterogeneity among the included studies with respect to infant characteristics and measurement time-points. Many studies had a cross-sectional rather than a longitudinal design which is not optimal for growth curve development. Further, there was inconsistent information on macronutrient intake, and so the impact of breast feeding was not examined. Nutritional information would be essential to the evaluation of growth. In particular, the intake of fat and total energy have been positively associated with increasing fat-mass, while protein with carbohydrate is related with increasing fat-free mass [23]. Data were obtained from infants who were treated with different nutritional protocols and encompassed a wide range of clinical characteristics. Therefore, while the compiled body composition data present a wide range of results, current available evidence is not sufficient to develop normative data for fat mass and fat-free mass in preterm and term infants. Furthermore, by 60 weeks PMA, an increasing number of infants reach the ADP device weight limitation of 8 kg. This leads to an underrepresentation of infants with higher weights, potentially confounding the average body composition values. The presented studies only provided average values, however using individual data points would have led to a wider variation of body composition measures. Moreover, considering that preterm infants are consistently shorter than term born infants, drawing conclusions from the absolute fat mass becomes less meaningful. Using the percent fat mass, fat mass index (FMI) and fat-free mass index (FFMI) would be more accurate. Most studies did not report FMI and FFMI. The calculation of these indices from mean fat mass, fat-free mass and length as abstracted from studies is not feasible, and so individual data are required.

In this study, the average fat mass obtained from ADP measurements was considerably lower in infants older than 50 weeks PMA compared to DXA measurements. As this occurs before 60 weeks PMA, it is unlikely that this is due to the ADP device weight limitation. These results suggest that DXA overestimates the fat mass for higher percent and absolute fat mass and that this finding is likely due to methodological differences. A similar finding was presented [11,24] and Fields et al. suggested that ADP might deliver more accurate estimations of body composition than DXA. Differences in body composition assessment between the two devices can be explained by the distinct techniques used by DXA and ADP. DXA measurements are based on X-ray attenuation characteristics of fat, lean tissues and bone mineral caused by photoelectric absorption, Compton (inelastic) scattering, and coherent (elastic) scattering [25]. However, it had been shown that differences in body composition measures exist between devices [11,26,27,28]. Other studies suggest that for high-quality DXA data, a scan acquisition protocol including clothing and positioning must be standardized, and special preterm and term infant validated scan modes and software are required [11,29,30]. Conversely, ADP is replacing underwater weighing or hydrodensitometry, and applies the principles of Boyle’s law, which describes the relationship between pressure and volume, for body composition measurement. ADP uses the infant’s total body density, which is derived from weight and accurately measured body volume, to calculate body composition and has the advantage of considering all compressible volumes such as the functional residual capacity of the lung, and provides high precision data [25,31,32]. Differences in DXA and ADP methods show that a comparison of body composition between cohorts should be performed with caution when different methods are used.

In summary, the current review provides an overview of available evidence regarding the body composition of preterm and term infants and highlights the limitations of this literature. Future studies should focus on longitudinal measurements in healthy preterm and term infants and consider factors that may influence this growth, to provide normative data as a clinical guide for growth outcomes.

## Figures and Tables

**Table 1:** Characteristics of included studies (ADP—air displacement plethysmography, DXA—dual X-ray energy absorptiometry, SGA—small for gestational age, AGA—appropriate for gestational age, LGA—large for gestational age, PMA—postmenstrual age).

	ADP		DXA	
	Preterm	Term	Preterm	Term
# of cohorts	32 *	91 *	14 *	19 *
<28 weeks PMA	2	n/a	2	n/a
28 to 31 + 6/7 weeks PMA	19	n/a	11	n/a
32 to 36 + 6/7 weeks PMA	14	n/a	5	n/a
# of longitudinal	16	34	10	5
# of cross-sectional	19	57	4	14
Total sample size infants	2855	22,410	1147	3542
Age at measurement (# of cohorts)				
<37 weeks PMA	8	n/a	7	n/a
37 to 41 + 6/7 weeks PMA	26	82	8	15
42 to 47 + 6/7 weeks PMA	5	14	2	3
48 to 56 + 6/7 weeks PMA	6	25	3	3
>57 weeks PMA	3	13	2	3
Studies that included maternal smoking history	No information	Yes: 20	No information	Yes: 5
Breast fed, formula, mix, no information	3, 2, 12, 23	115, 4, 23, 53	4, 5, 5, 6	2, 0, 5, 13
SGA, AGA, LGA, no information	2, 28, 0, 5	2, 66, 2, 21	3, 14, n/a, n/a	3, 15, 2, 2

* Columns present number of cohorts, some studies had data of multiple cohorts available.

## Supplementary Materials

The following are available online at https://www.mdpi.com/2072-6643/12/2/288/s1, Figure S1: Body composition (percent fat mass, fat mass, fat-free mass) of preterm and term infants, Figure S2: Anthropometric data (weight, length, head circumference) for postmenstrual age for preterm and term infants, Figure S3: Comparison of body composition data (percent fat mass, fat mass, fat-free mass, weight, length, head circumference) between studies which matched the World Health Organization (WHO) Multicentre Growth Reference Study (MGRS) with other studies included in this review (blue—all studies, red—WHO MGRS), dotted lines indicate body composition at 40 and 52 weeks postmenstrual age; Figure S4: Body composition (percent fat mass, fat mass, fat-free mass) for postmenstrual age in preterm and term infants measured with dual energy X-ray absorptiometry (DXA), Figure S5: Comparison of ADP and DXA measurements in preterm and term infants for postmenstrual age, Table S1: Overview of reviewed articles with body composition measurements using air displacement plethysmography (APD) in preterm and term infants, Table S2: Overview of reviewed articles with body composition measurements using dual energy X-ray absorptiometry (DXA) in preterm and term infants, Table S3: Percent fat mass percentiles for term infants measured with ADP, Table S4: Fat mass percentiles for term infants measured with ADP, Table S5: Fat-free mass percentiles for term infants measured with ADP.
