# Supplementary material for: Fat and Fat-Free Mass of Preterm and Term Infants from Birth to Six Months: A Review of Current Evidence
Source: Nutrients. 2020 Jan 21;12(2):288. doi: 10.3390/nu12020288 (PMC7070317; doi:10.3390/nu12020288)
Supplement: Supplementary file 1 [file nutrients-12-00288-s001.zip › Suppl-1 ADP 2019-12-14.docx]

**Table S1:** Overview of reviewed articles with body composition measurements using air displacement plethysmography (APD) in preterm and term infants

AGA: appropriate for gestational age, BF: breast feeding, CMA: cow milk allergy, GD: gestational diabetes, GWG: gestational weight gain, LGA: large for gestational age, MF: mixed feeding, NGT: normal glucose tolerance, NW: normal weight, SGA: small for gestational age, VLBW: very low birth weight

| **Name** | **Year of study** | **Country** | **Type of study** | **Number, gestational age, Birth weight** | **Age at study (PMA)** | **Info** |
| --- | --- | --- | --- | --- | --- | --- |
| Gianni 2009 [1] | 2007-2008 | Italy | cross-sectional | n=67, 31 wks, 1140g | 39.4 wks | SGA, MF, |
|  |  |  |  | n=132, 39 wks, 2430g | 39 wks | SGA, BF |
| Roggero 2009 [2] | 2006-2007 | Italy | cross-sectional | n=110, 30 wks, 1120g | 39.5 wks |  |
|  |  |  |  | n=87, 39 wks, 3200g | 39 wks | BF |
| Ramel 2011 [3] | 2008-2009 | USA | longitudinal | n=26, 32 wks, 1720g | 40, 53 wks | MF |
|  |  |  |  | n=97, 40 wks, 3540g | 40, 53 wks | MF |
| Roggero 2008 [4] | 2006 | Italy | longitudinal | n=53, 31 wks, 1360g | 40, 44, 48, 52 wks | Preterm formula fed before discharge, Nutrient-enriched postdischarge formula-fed after term |
| Villar 2017  [5] | 2011-2016 |  | cross-sectional | n=91, 36 wks | 36.4 wks | MF |
|  |  |  |  | n=928, 39 wks, 3240g | 39.5 wks | MF |
| Liotto 2017  [6] | 2012-2016 | Italy | cross-sectional | n=585, 32 wks, 1750g | 40 wks |  |
|  |  |  |  | n=654, 39 wks, 3220g | 39 wks |  |
| Villela 2018  [7] | 2012-2016 | Brazil | longitudinal | n=67, 30 wks, 1300g | 40, 44, 52, 60 wks | AGA |
|  |  |  |  | n=25, 32 wks, 1270g | 40, 44, 52, 60 wks | SGA |
| Scheurer 2017 [8] | 2008-2009 | USA | longitudinal | n=20, 32 wks, 1840g | 40, 54 wks | AGA |
|  |  |  |  | n=51, 40 wks | 40, 54 wks | AGA |
| Henriksson 2017 [9] | 2007-2008 | Sweden | longitudinal | n=26, 40 wks | 41, 52 wks | MF |
| Larcade 2016 [10] | 2014-2015 | France | cross-sectional | n=155, 29 wks, 1210g | 37.5 wks |  |
| Ramel 2014 [11] | 2010-2012 | USA | cross-sectional | n=98, 34 wks, 2270 g | 34.3 wks |  |
| Alexandre-Gouabau 2018 [12] | 2011-2016 | France | cross-sectional | n=15, 30 wks, 1610g | 37 wks | Slower growth rate (−1.54 ± 0.42 *Z*-score) |
|  |  |  |  | n=11, 31 wks, 1200g | 38.3 wks | Faster growth rate (−0.48 ± 0.19 *Z*-score) |
| Dahly [13] | 2008-2011 | Ireland | cross-sectional | n=14, 40 wks, 3260g | 40 wks |  |
| Liotto 2018  [14] | 2014-2016 | Italy | longitudinal | n=50, 39 wks, 3270g | 39, 48, 56 wks | Formula A (composition of formula A vs. formula B were for energy (65 vs. 68 kcal/100 mL), protein (1.2 vs. 1.7 g/100 mL), protein-to-energy ratio (1.9 vs. 2.5 g/100 kcal), carbohydrates  (8 vs. 7.1 g/100 mL), fat (3.1 vs. 3.5 g/100 mL), AGA |
|  |  |  |  | n=50, 38 wks, 3270g | 39, 48, 56 wks | Formula B, AGA |
|  |  |  |  | n=50, 39 wks, 3180g | 39, 48, 56 wks | BF, AGA |
| Frondas-Chauty 2018 [15] | 2008-2013 | France | cross-sectional | n=19, 28 wks, | 39.2 wks | Ages and stages Questionnaire (ASQ) <186 |
|  |  |  |  | n=25, 30 wks | 38 wks | ASQ 186-220 |
|  |  |  |  | n=113, 30 wks | 37.6 wks | ASQ > 220 |
| Gallagher 2018 [16] | 2013-2015 | USA | cross-sectional | n=105, 39 wks, 3370g | 39 wks | Lifestyle intervention Infants, focused on controlling gestational weight gain through nutrition and activity behaviors |
|  |  |  |  | n=105, 39 wks, 3240g | 39 wks | usual obstetric care infants |
| Morlacchi 2018 [17] | 2014-2016 | Italy | longitudinal | n=17, 29 wks, 1210g | 36.6, 40 wks | Fortified human milk |
|  |  |  |  | n=15, 30 wks, 1290g | 37.5, 40 wks | Preterm Formula |
| Rudolph 2017 [18] | unknown | USA | longitudinal | n=48, 40 wks, 3360g | 41.9, 56.5 wks | BF |
| Moore 2017 [19] |  |  | cross-sectional | n=813, 40 wks, 3300g | 40.2 wks |  |
| Paviotti 2017 [20] | 2013-2015 | Italy | cross-sectional | n=42, 29 wks, 1120g | 40.2 wks | Preterm |
| Andersen 2011[21] | 2009-2010 | Ethiopia | cross-sectional | male: n=167,  female: n=183; 39 wks | 39 wks |  |
| Carberry 2010 [22] | 2008-2009 | Australia | longitudinal | male: n=25, 40 wks, | 40, 46, 42.7, 59.4 wks | Non-obese mothers |
|  |  |  |  | female: n=20, 40 wks | 40, 46, 52.7, 59.4 wks | Non-obese mothers |
| Dong 2018[23] | 2014-2015 | China | cross-sectional | n=60, 40 wks, 3300g | 52 wks | CMA |
|  |  |  |  | n=60, 40 wks, 3400g | 52 wks | Control |
| Baker 2017[24] | 2010-2014 | USA | longitudinal | n=10, 39 wks, 3320g | 39.6, 57 wks | Rapid Postnatal Gain in adiposity: higher %FM and lower %FFM at 5 months compared to the mean |
|  |  |  |  | n=13, 39 wks, 3300 | 39.6, 58 wks | Lower postnatal gain in adiposity: lower %FM and higher %FFM at 5 months compared to the mean |
| Perng 2017 [25] | 2009-2014 | USA | longitudinal | n=1027, 40 wks | 40, 60 wks |  |
| Eriksson 2009 [26] | 2007-2008 | Sweden | longitudinal | Female: n=53, 40 wks, 3530g | 41.3, 52.4 wks | MF |
|  |  |  |  | male: n=55, 40 wks, 3800g | 41, 52.1 wks | MF |
| Fields 2009 [27] | 2006-2008 | USA | longitudinal | female: n=64, 39 wks, 3500g | 43.3, 51.3, 63.3 wks | No GD, AGA |
|  |  |  |  | male: n=53, 39 wks, 3350g | 43.3, 51.3, 63.3 wks | No GD, AGA |
| Fields 2011 [28] | unknown | USA | longitudinal | female: n=76, 39 wks, 3030g | 39.4, 40.3, 41.3, 43.3, 47.3, 51.3, 55.3, 59.3, 63.3 wks | Breastfed, AGA |
|  |  |  |  | male: n=84, 39 wks, 3410g | 39.4, 40.3, 41.3, 43.3, 47.3, 51.3, 55.3, 59.3, 63.3 wks | Breastfed, AGA |
| Fields 2012[29] | unknown | USA | cross-sectional | N=84, 40 wks, 3442g | 64 wks | No GD |
| Hull 2008[30] | unknown | USA | cross-sectional | n=33, 40 wks, 3430g | 42 wks | normal |
|  |  |  |  | n=39, 39 wks, 3370g | 42 wks | obese mothers |
| Lingwood 2011[31] | 2007-2009 | Australia | longitudinal | n=77, 40 wks, 3600g | 40.2, 46, 52.7, 59.4 wks | NW mothers |
| Olhager 2013 [32] | 2008-2009 | Sweden | longitudinal | n=29, 35 wks, 2440g | 35.5, 38.7 wks | preterm |
|  |  |  |  | n=29, 39 wks, 3190g | 39.7 wks | term |
| Roggero 2010[33] | 2006-2007 | Italy | longitudinal | n=28, 39 wks, 3170g | 38.8, 38.9, 39.1, 39.2, 39.4 wks | BF, AGA |
| Stanfield 2012 [34] | 2007-2008 | UK | cross-sectional | n=30, 40 wks, 3560g | 47.4 wks | no gestational diabetes,White British |
|  |  |  |  | n=30, 39 wks, 3060g | 47.7 wks | no gestational diabetes, South Africa |
| Admassu 2017[35] | 2009-2014 | Ethiopia | cross-sectional | n=268, 39 wks, 3100g | 39.1 wks |  |
| Gianni 2016[36] | 2014-2015 | Italy | longitudinal | n=122, 35 wks, 2420g | 36, 39.4 wks |  |
|  |  |  |  | n=42, 40 wks, 3060g | 40.1 wks | BF, AGA |
| Kadakia 2017[37] | 2011-2014 | USA | cross-sectional | n=105, 39 wks, 3500g | 39.8 wks | NGT |
| Sauder 2017 [38] | 2009-2014 | USA | longitudinal | n=348, 39 wks, 3160g | 39.6, 59.5 wks |  |
| Mckenzie 2017[39] | 2015-2016 | Australia | cross-sectional | n=147, 39wks, 3430g | 39.4 wks | No GD |
| Abera 2017[40] | 2009-2012 | Ethiopia | cross-sectional | n=227, 40 wks, 3100g | 40 wks |  |
| Toro-Ramos 2017 [41] |  | USA | cross-sectional | n=42, 40 wks, 3140g | 40 wks |  |
| Cauble 2017 [42] | unknown | unknown | longitudinal | n=95, 39 wks, 3500g | 39.3 wks | No GD |
|  |  |  |  | n=63, 39 wks, 3540g | 56 wks | No GD |
| Chen 2018 [43] | 2009-2010 | Singapore | cross-sectional | n=251, 40 wks, 3200g | 42.2 wks |  |
| Chia 2018 [44] | 2009-2010 | Singapore | cross-sectional | n=313, 40 wks, 3090g | 40 wks |  |
| Lemas 2016 [45] | 2012-2015 | USA | cross-sectional | n=18, 40 wks, 3550g | 41.7 wks | BF, no diabetes, normal weight |
|  |  |  |  | n=12, 40 wks, 3460g | 41.7 wks | BF, no diabetes obese mothers |
| Breij 2016 [46] | 2013- | Netherlands | longitudinal | female: n=93, 40 wks | 43.9, 51.9, 63.9 wks |  |
|  |  |  |  | male: n=104, 40 wks, | 43.7, 51.7, 63.7 wks |  |
| Paley 2016 [47] | 2006-2009 | USA | cross-sectional | female: n=23, 39 wks, 3210g | 39.5 wks | non diabetes Afro-American |
|  |  |  |  | male: n=21, 39 wks, 3140g | 39.5 wks | non diabetes Afro-American |
|  |  |  |  | female: n=16, 40 wks, 3160 g | 39.8 wks | non diabetes Asian |
|  |  |  |  | male: n=14, 40 wks, 3560g | 40.5 wks | non diabetes Asian |
|  |  |  |  | female: n=98, 40 wks, 3400g | 39.8 wks | non diabetes Caucasian |
|  |  |  |  | male: n=88, 40 wks, 3550g | 39.8 wks | non diabetes Caucasian |
|  |  |  |  | female: n=32, 39 wks, 3200g | 39.5 wks | non diabetes Hispanic |
|  |  |  |  | male: n= 40, 40 wks, 3490g | 39.8 wks | non diabetes Hispanic |
| Hawkes 2016 [48] | 2008-2011 | Ireland | longitudinal | male: n=537, 40 wks, 3570g | 40.4, 49.5 wks |  |
|  |  |  |  | female: n=526, 40 wks, 3470g | 40.4, 49.5 wks |  |
| McLeod 2016 [49] | 2009 | Australia | cross-sectional | n=20, 27 wks, 1020g | 37.7 wks | BF, Intervention group |
|  |  |  |  | n=20, 27 wks, 1010g | 37.8 wks | BF, routine practice group |
| Ramel 2016 [50] | 2010-2014 | USA | cross-sectional | n=218, 34 wks, 2190g | 33.9 wks | AGA |
| Gianni, Consonni 2016 [51] | 2015-2016 | Italy | cross-sectional | male: n=147, 2430g; female: n=137, 2400g; 35 wks | 40 wks | AGA |
| Kizirian 2016 [52] | 2011-2013 | Australia | longitudinal | n=28, 39 wks, 3400g | 39.5, 51.4 wks | overweight mothers (prepragnancy BMI ≥ 30), Low Glycemic index (51 ± 1) |
|  |  |  |  | n=29, 40 wks, 3600g | 39.5, 51.9 wks | overweight (BMI prepragnancy ≥ 30), high fiber group |
| Josefson 2016 [53] |  |  | cross-sectional | n=168, 40 wks, 3470g | 39.6 wks | NGT |
| Scheurer 2016 [54] | 2011-2012 | USA | longitudinal | n=12, 27wks, 930g | 39.1, 58.2 wks | AGA, 1-5 days |
|  |  |  |  | n=8, 24 wks, 680g | 40.2, 56.6 wks | AGA; > 5 days |
|  |  |  |  | n=30, 29 wks, 1220g | 37.1, 57.6 wks | AGA,0 days |
| Sauder 2016 [55] | 2009-2014 | USA | longitudinal | n=30, 39 wks, 2940g | 39.5, 60.7 wks | Non-users of multivitamins |
|  |  |  |  | n=149, 30 wks, 3090g | 39.7, 61 wks | 1 quartile (weeks of daily use) of Multivitamin use |
|  |  |  |  | n=156, 39 wks, 3050g | 39.6, 61 wks | 2 quartile of multivitamin use |
|  |  |  |  | n=142, 40 wks, 3180g | 40.1, 61.4 wks | 3 quartiles of multivitamin use |
|  |  |  |  | n=142, 40 wks, 3200g | 40, 61.2 wks | 4 quartiles of multivitamin use |
| Breij 2017[56] | 2013 | Netherlands | longitudinal | male: n=119, 3360g; female: n=84, 3250g; 40 wks | 44, 52, 64 wks |  |
| Kizirian 2016 (2) [57] | unknown | Australia | cross-sectional | male: n=48, 39 wks, 3400g | 39.4 wks | Overweight m |
|  |  |  |  | female: n=12, 40 wks, 3500g | 40 wks | Overweight m |
| Crume 2016 [58] | unknown | USA | cross-sectional | n=511, 40 wks, 3280g | 39.8 wks |  |
| Shapiro 2016 [59] | 2010-2014 | USA | cross-sectional | n=647, 39 wks, 3230g | 39.8 wks | Healthy eating index < 57 (low scores respresent poorer diet quality) |
|  |  |  |  | n=432, 40 wks, 3300g | 40 wks | Healthy Eating Index > 57 |
| Tinius 2016 [60] | 2013-2014 | USA | cross-sectional observational | n=15, 40 wks, 3340g | 39.6 wks | Obese inactive |
|  |  |  |  | n=15, 39 wks, 3230g | 39.6 wks | Obese active |
| Barbour [61] | 2008-2014 | USA | cross-sectional observational | n=28, 40 wks, 3320g | 40 wks |  |
| Starling 2015 [62] | 2010-2013 | USA | cross-sectional observational | n=826, 40 wks, 3150g | 40.2 wks |  |
| Lemas 2015 [63] | 2009-2014 | USA | cross-sectional observational | n=405, 40 wks, 3260 g | 40 wks | normal weight |
|  |  |  |  | n=200, 40 wks, 3310 | 40 wks | overweight |
|  |  |  |  | n=155, 40 wks, 3360g | 40wks | obese |
| Harrod 2015 [64] | 2010-2013 | USA | longitudinal | n=47, 39wks, 3270g | 39.3 wks | yes smoking |
|  |  |  |  | n=623, 40 wks, 3270g | 39,7 wks | MF, no smoking |
| Henriksson 2015 [65] | 2008-2011 | Sweden | cross-sectional | female: n=99, 40 wks, 3540g | 41 wks | MF |
|  |  |  |  | male: n=110, 40 wks, 3660g | 42.1 wks | MF |
| Crume 2015 [66] | unknown | USA | cross-sectional | n=805, 39.4 | 39.5 wks |  |
| Roggero 2015 [67] | 2011-2012 | Italy | cross-sectional | n=50, 30 wks, 1210g | 41 wks | Preterm, AGA |
|  |  |  |  | n=34, 39 wks, 3370g | 39.4 wks | BF, AGA |
| Wibæk 2015 [68] | 2012 | Ethiopia | longitudinal | total n=101, female: 39 wks, 2990g; | 39, 45.6, 49.6, 53.6, 57.9, 64.7 wks |  |
|  |  |  |  | male: 39 wks, 3150g | 39, 45.8, 49.6, 53.9, 57.9, 64.5 wks |  |
| Breij 2015 [69] | 2012-2013 | Netherlands | cross-sectional | n=194, 39 wks, 3380g | 39.4 wks |  |
| De Cunto [70] | 2012-2014 | Italy | cross-sectional | n=21, 35 wks, 2430g | 43 | surgery |
|  |  |  |  | n=21, 35 wks, 2420g | 43 | no surgery |
| Henriksson 2014 [71] | 2007-2010 | Sweden | cross-sectional | n=57, 40 wks, 3360g | 40.7 wks | GWG (gestational weight gain) below IOM (institute of medicine) recommendations |
|  |  |  |  | n=114, 40 wks, 3590g | 41 wks | GWG within IOM recommendations |
|  |  |  |  | n=141, 40 wks, 3760g | 41.3 wks | GWG above IOM recommendations |
| McLeod 2015 [72] | 2007 | Australia | cross-sectional | n=12, 29 wks, 1400g | 39 wks |  |
| Donnelley 2014 [73] | 2010 | Australia | cross-sectional | n=61, 40 wks | 39.7 wks | LGA |
|  |  |  |  | n=475, 40 wks |  | AGA |
| Gianni 2014 [74] | 2007-2008 | Italy | longitudinal, observational | male: n=34, 30 wks, 1150g | 39.3 wks | MF,Preterm males |
|  |  |  |  | female: n=29, 30 wks, 1150g | 39.4 wks | MF, Preterm females |
|  |  |  |  | male: n=32, 39 wks, 3250g | 39.4 wks | term males, breastfed |
|  |  |  |  | female: n=29, 39 wks, 3250g | 39.2 wks | BF, term females |
| Simon 2014 [75] |  | France | prospective observational | n=47, 30 wks | 37.2 wks | Tertile 3 (Comparison of perinatal characteristics and growth during neonatal hospitalization between tertiles  of FM percentage assessed at discharge) |
|  |  |  |  | n=47, 32 wks | 37.3 wks | Tertile 2 |
|  |  |  |  | n=47, 32 wks |  | Tertile 1 |
| Gianni 2014 (2) [76] | 2012-2013 | Italy | longitudinal, observational | n=64, 39 wks, 3170g | 39.6, 54.7 wks | Breastfed |
|  |  |  |  | n=61, 39 wks, 3200g | 39.9, 54.7 wks | Formula |
| Josefson 2014 [77] | unknown | USA | cross-sectional | n=38, 40 wks, 3380g | 40 wks | NW mothers |
|  |  |  |  | n=23, 40 wks, 3600g | 40 wks | Obese Mothers |
| Hull 2014 [78] | unknown | USA | cross-sectional | n=20, 40 wks, no birthweight | 40.1 wks | Underweight M |
|  |  |  |  | n=252, 40 wks, no birthweight | 39.9 wks | Normal m |
|  |  |  |  | n=59, 39 wks, no birthweight | 39.5 wks | Overweight m |
|  |  |  |  | n=40, 39 wks, no birthweight | 39.7 wks | obese m |
| Harrod 2014 [79] | 2010-2013 | USA | longitudinal | n=826, 40 wks, 3290g | 39.7 wks |  |
| Harrod 2014 (2) [80] | 2010-2013 | USA | longitudinal | n=85, 39 wks, 3040g | 39.3 wks | Prenatal smoking yes |
|  |  |  |  | n=831, 39 wks, 3280g | 39.6 wks | no prenatal smoking |
| Pereira-da-Silva 2014 [81] | 2010-2011 | Portugal | Cross section | m+f: n=100, 40 wks, 3360g | 39.9 wks |  |
| Henriksson 2014 (2) [82] | 2008-2010 | Sweden | longitudinal | m+f: n=207, 39 wks, no birthweight | 40, 51 wks | Mixed feeding (BM and formula) |
| Andersen 2013 [83] | unknown | Ethiopia | longitudinal | n=348, male: 3110g, female: 3000g, 39 wks | 39.1, 45, 49, 53, 57, 63 wks |  |
| Au 2013 [84] | 2010 | Australia | cross-sectional | n=67, 40 wks, 3270 | 39.7 wks | GDM |
|  |  |  |  | n=532, 40 wks, 3440g | 39.7 wks | Normal glucose tolerance |
| Carberry 2013 [85] | 2010 | Australia | cross-sectional | n=550, 39 wks, 3430g | 39.4 wks |  |
| Josefson 2013 [86] | unknown | USA | cross-sectional | n=27, 40 wks, 3390g | 40 wks | Gestational weight gain within IOM Guidelines 25-35 lb |
|  |  |  |  | n=11, 40 wks, 3830g | 40.2 wks | excessive gain |
| Pfister 2013 [87] | unknown | USA | longitudinal | n=16, 30 wks, 1530g | 40.8 wks | MF, AGA |
| Carberry 2013 (2) [88] | 2010 | Australia | cross-sectional | n=581, 40 wks, 3430g | 39.6 wks |  |
| Josefson 2013 (2) [89] |  | USA | cross-sectional | n=38, 40 wks, 3380g | 40 wks | NW mothers |
|  |  |  |  | n=23, 40 wks, 3600g | 40 wks | obese m |
| Paviotto 2013 [90] | 2011-2012 | Italy | longitudinal, observational | n= 36, 37 wks, 2440g | 36.7, 39, 40.9 wks | MF, twins |
|  |  |  |  | n=36, 37 wks, 2450g | 37.2, 39.2, 41.1 wks | MF, singleton |
| Meyers 2013 [91] | 2010-2011 | USA | cross-sectional | n=39, 33 wks, 1590g | 36.7 wks | Total parenteral nutrition (TPN), AGA |
|  |  |  |  | n=22, 34 wks, 2270g | 36.8 wks | No TPN, AGA |
| Deierlein 2012 [92] | unknown | USA | cross-sectional | male: n=68, 39 wks | 39.4 wks |  |
|  |  |  |  | female: n=60, 39 wks | 39.2 wks |  |
| Eriksson 2012 [93] | unknown | Sweden | longitudinal | female: n=21, 40 wks, 3710g | 41, 52 wks |  |
|  |  |  |  | male: n=23, 40 wks, 3810g | 41.2, 52 wks |  |
| Hull 2011 [94] | unknown | USA | cross-sectional | n=210, 40 wks, no birthweight | 40 wks | Normal mothers (BMI 18.5 – 24.9 kg/m2) |
|  |  |  |  | n=59, 39 wks, no birthweight | 39.6 wks | Overweight mothers (BMI 25 – 29.9 kg/m2) |
|  |  |  |  | n=37, 39 wks, no birthweight | 39.7 wks | obese mothers (BMI >30.0 kg/m2) |
| Taroni 2012 [95] | unknown | Italy | cross-sectional | n=25, 30 wks, 1170g | 42.5 wks |  |
|  |  |  |  | n=10, 38 wks, 3380g | 41.6 wks |  |
| Andres 2012 [96] | 2006-2007 |  | cross-sectional | n=46, 39 wks, 3410g | 41 wks | Lean mothers (Prepragnancy BMI 22.0 ± 1.6) |
|  |  |  |  | n=19, 39 wks, 3650g | 41.1 wks | BF, Overweight mothers (prepragnancy BMI: 27.1 ±1.6) |
| Roggero 2010 [97] | unknown | unknown | longitudinal | female: n=35, 39 wks, 2980g | 39.3, 41.2, 43.2, 47.2, 51.2, 55.2, 59.2, 63.2 wks | BF, no GD |
|  |  |  |  | male: n=24, 39 wks, 3200g | 39.3, 41.2, 43.2, 47.2, 51.2, 55.2, 59.2, 63.2 wks | BF, no GD |
| Law 2011 [98] | 2009-2011 | USA | cross-sectional | n=26, 38 wks, 2350g | 37.6 wks | Estimated fetal weight less than the 10th percentile (followed with ultrasound) |
|  |  |  |  | n=26, 38,1 wks, 2680g | 38.1 wks | Abdominal Circumference less than the fifth percentile |
|  |  |  |  | n=44, 39 wks, 3270g | 38.6 wks | Normal biometry |
| Algotar 2017 [99] | unknown | USA | longitudinal | n=22, 32 wks, 1700g | 37.5, 41 wks |  |
| Piemontese 2018 [100] | 2016-2017 | Italy | longitudinal | n=24, 30 wks, 1210g | 40 wks | VLBW |
|  |  |  |  | n=49, 30 wks, 1270g | 40 wks | VLBW |
| Moyer-Mileur 2009[101] | Unknown | USA | Cross section | n=5, 38 wks, 2540g | 38.1 wks | SGA |
|  |  |  |  | n=38, 38 wks, 3320g | 38.1 wks | AGA |
|  |  |  |  | n=4, 38 wks, 4520g | 38.1 wks | LGA |
| Roggero 2009 [33] | 2006-2007 | Italy | Cross section | n=56, 39 wks, 3260g | 39.4 wks | 28 male, 28 female, breastfed |
|  |  |  |  | n=96, 39 wks, 3120g | 39.5 wks | 51 male, 45 female, breastfed |
|  |  |  |  | n=56, 39 wks, 3020g | 39.6 wks | 26 male, 30 female, breastfed |
|  |  |  |  | n=54, 39 wks, 2940g | 39.8 wks | 30 male, 24 female, breastfed |
|  |  |  | longitudinal | n=28, 39 wks, 3170g | 38.6, 38.8, 38.9, 39, 39.2 wks | 13 male, 15 female, breastfed |
| Anderson 2010 [102] | 2006-2007 | USA | Cross section | n=65, 40 wks, 3500g | 51.5 wks | Exclusive breastfeeding |
|  |  |  |  | n=20, 39 wks, 3410g | 51.1 wks | Mixed feeding (BM and formula) |
|  |  |  |  | n=11, 39 wks, 3390g | 51 wks | Exclusive formula feeding |
| Gianni 2012 [103] | Unknown | Italy | Longitudinal | n=49, 35 wks, 2500g | 40, 44, 52 wks | Late preterm, mixed feeding |
|  |  |  |  | n=40, 39, wks, 3070g | 39.3, 43.2, 51.2 wks | Full-term, healthy, BF |
| Lee 2012 [104] | 2004-2009 | USA | cross-sectional | female: n=164, 39 wks, 3300g | 38.7 wks | Non-diabetic pregnancies |
|  |  |  |  | male: n=160, 39wks, 330g | 38.7 wks | Non-diabetic pregnancies |
|  |  |  |  | n=25, 38 wks, 3620g | 38.2 wks | Diabetic pregnancies |
| Aris 2013 [105] | 2009-2011 | Singapore | cross-sectional | n=62, 39 wks, 3200g | 38.8 wks |  |
|  |  |  |  | n=26, 39wks, 3400g | 38.8 wks |  |
|  |  |  |  | n=174, 39 wks, 3200g | 38.8 wks |  |
| Markovic 2015 [106] | 2011-2012 | Australia | cross-sectional | n=65, 40 wks, 3430g | 39.7 wks | Mothers with low glycemic Index diet (target GI <50) |
|  |  |  |  | n=60, 39 wks, 3380g | 39.5 wks | Mothers with high fiber diet (target GI > 60) |
| Tinius 2015 [107] | 2013-2014 | USA | cross-sectional | n=18, 39,2 wks, no birthweight | 39.2 wks | lean mothers (pre-pregnancy BMI: 21.5 ± 1.9) |
| Kadakia 2016 [108] | 2011-2013 | USA | cross-sectional | n=112, 40 wks, 3510g | 39.7 wks |  |
| Hawkes 2011 [109] | 2008-2010 | unknown | cross-sectional | n=45, 37 wks, 2960g | 37.5 wks |  |
|  |  |  |  | n=243, 39 wks, 3330g | 39.4 wks |  |
|  |  |  |  | n=455, 41 wks, 3540g | 41 wks |  |
| Tint 2016 [110] | 2009-2011 | Singapore | Longitudinal | n=94, 39 wks, 3100g | 40.3 wks | BF, measurements were taken at 2 weeks |
|  |  |  |  | n=46, 39 wks, 3150g | 40.2 wks |  |

1. Gianni, M.L.; Roggero, P.; Taroni, F.; Liotto, N.; Piemontese, P.; Mosca, F. Adiposity in small for gestational age preterm infants assessed at term equivalent age. *Archives of Disease in Childhood-Fetal and Neonatal Edition* **2009**, *94*, F368-F372, doi:10.1136/adc.2008.153163.

2. Roggero, P.; Gianni, M.L.; Amato, O.; Orsi, A.; Piemontese, P.; Morlacchi, L.; Mosca, F. Is term newborn body composition being achieved postnatally in preterm infants? *Early Human Development* **2009**, *85*, 349-352, doi:10.1016/j.earlhumdev.2008.12.011.

3. Ramel, S.E.; Gray, H.L.; Ode, K.L.; Younge, N.; Georgieff, M.K.; Demerath, E.W. Body Composition Changes in Preterm Infants Following Hospital Discharge: Comparison With Term Infants. *Journal of Pediatric Gastroenterology and Nutrition* **2011**, *53*, 333-338, doi:10.1097/MPG.0b013e3182243aa7.

4. Roggero, P.; Gianni, M.L.; Amato, O.; Orsi, A.; Piemontese, P.; Puricelli, V.; Mosca, F. Influence of protein and energy intakes on body composition of formula-fed preterm infants after term. *Journal of Pediatric Gastroenterology and Nutrition* **2008**, *47*, 375-378, doi:10.1097/MPG.0b013e3181615cba.

5. Villar, J.; Puglia, F.A.; Fenton, T.R.; Ismail, L.C.; Staines-Urias, E.; Giuliani, F.; Ohuma, E.O.; Victora, C.G.; Sullivan, P.; Barros, F.C., et al. Body composition at birth and its relationship with neonatal anthropometric ratios: the newborn body composition study of the INTERGROWTH-21st project. *Pediatric Research* **2017**, *82*, 305-316, doi:10.1038/pr.2017.52.

6. Liotto, N.; Roggero, P.; Bracco, B.; Menis, C.; Morniroli, D.; Perrone, M.; Giannì, M.L.; Mosca, F. Can Basic Characteristics Estimate Body Composition in Early Infancy? *J Pediatr Gastroenterol Nutr* **2018**, *66*, e76-e80, doi:10.1097/MPG.0000000000001758.

7. Villela, L.D.; Méio, M.D.B.B.; Gomes Junior, S.C.S.; de Abranches, A.D.; Soares, F.V.M.; Moreira, M.E.L. Body composition in preterm infants with intrauterine growth restriction: a cohort study. *J Perinat Med* **2018**, *46*, 804-810, doi:10.1515/jpm-2017-0175.

8. Scheurer, J.M.; Zhang, L.; Gray, H.L.; Weir, K.; Demerath, E.W.; Ramel, S.E. Body Composition Trajectories From Infancy to Preschool in Children Born Premature Versus Full-term. *Journal of Pediatric Gastroenterology and Nutrition* **2017**, *64*, E147-E153, doi:10.1097/mpg.0000000000001494.

9. Henriksson, H.; Eriksson, B.; Forsum, E.; Flinke, E.; Henriksson, P.; Lof, M. Longitudinal assessment of body composition in healthy Swedish children from 1 week until 4 years of age. *Eur J Clin Nutr* **2017**, 10.1038/ejcn.2017.125, doi:10.1038/ejcn.2017.125.

10. Larcade, J.; Pradat, P.; Buffin, R.; Leick-Courtois, C.; Jourdes, E.; Picaud, J.C. Estimation of Fat-free Mass at Discharge in Preterm infants Fed With Optimized Feeding Regimen. *Journal of Pediatric Gastroenterology and Nutrition* **2017**, *64*, 115-118, doi:10.1097/mpg.0000000000001261.

11. Ramel, S.E.; Gray, H.L.; Davern, B.A.; Demerath, E.W. Body composition at birth in preterm infants between 30 and 36 weeks gestation. *Pediatr Obes* **2015**, *10*, 45-51, doi:10.1111/j.2047-6310.2013.00215.x.

12. Alexandre-Gouabau, M.C.; Moyon, T.; Cariou, V.; Antignac, J.P.; Qannari, E.M.; Croyal, M.; Soumah, M.; Guitton, Y.; David-Sochard, A.; Billard, H., et al. Breast Milk Lipidome Is Associated with Early Growth Trajectory in Preterm Infants. *Nutrients* **2018**, *10*, 28, doi:10.3390/nu10020164.

13. Dahly, D.L.; Li, X.; Smith, H.A.; Khashan, A.S.; Murray, D.M.; Kiely, M.E.; O'B Hourihane, J.; McCarthy, F.P.; Kenny, L.C.; Kearney, P.M., et al. Associations between maternal lifestyle factors and neonatal body composition in the Screening for Pregnancy Endpoints (Cork) cohort study. *Int J Epidemiol* **2018**, *47*, 131-145, doi:10.1093/ije/dyx221.

14. Liotto, N.; Orsi, A.; Menis, C.; Piemontese, P.; Morlacchi, L.; Condello, C.C.; Gianni, M.L.; Roggero, P.; Mosca, F. Clinical evaluation of two different protein content formulas fed to full-term healthy infants: a randomized controlled trial (vol 18, 59, 2018). *Bmc Pediatrics* **2018**, *18*, 1, doi:10.1186/s12887-018-1109-8.

15. Frondas-Chauty, A.; Simon, L.; Flamant, C.; Hanf, M.; Darmaun, D.; Roze, J.C. Deficit of Fat Free Mass in Very Preterm Infants at Discharge is Associated with Neurological Impairment at Age 2 Years. *Journal of Pediatrics* **2018**, *196*, 301-304, doi:10.1016/j.jpeds.2017.12.017.

16. Gallagher, D.; Rosenn, B.; Toro-Ramos, T.; Paley, C.; Gidwani, S.; Horowitz, M.; Crane, J.; Lin, S.; Thornton, J.C.; Pi-Sunyer, X. Greater Neonatal Fat-Free Mass and Similar Fat Mass Following a Randomized Trial to Control Excess Gestational Weight Gain. *Obesity* **2018**, *26*, 578-587, doi:10.1002/oby.22079.

17. Morlacchi, L.; Roggero, P.; Giannì, M.L.; Bracco, B.; Porri, D.; Battiato, E.; Menis, C.; Liotto, N.; Mallardi, D.; Mosca, F. Protein use and weight-gain quality in very-low-birth-weight preterm infants fed human milk or formula. *Am J Clin Nutr* **2018**, *107*, 195-200, doi:10.1093/ajcn/nqx001.

18. Rudolph, M.C.; Young, B.E.; Lemas, D.J.; Palmer, C.E.; Hernandez, T.L.; Barbour, L.A.; Friedman, J.E.; Krebs, N.F.; MacLean, P.S. Early infant adipose deposition is positively associated with the n-6 to n-3 fatty acid ratio in human milk independent of maternal BMI. *International Journal of Obesity* **2017**, *41*, 510-517, doi:10.1038/ijo.2016.211.

19. Moore, B.F.; Sauder, K.A.; Starling, A.P.; Ringham, B.M.; Glueck, D.H.; Dabelea, D. Exposure to secondhand smoke, exclusive breastfeeding and infant adiposity at age 5months in the Healthy Start study. *Pediatric Obesity* **2017**, *12*, 111-119, doi:10.1111/ijpo.12233.

20. Paviotti, G.; De Cunto, A.; Zennaro, F.; Boz, G.; Travan, L.; Cont, G.; Bua, J.; Demarini, S. Higher growth, fat and fat-free masses correlate with larger cerebellar volumes in preterm infants at term. *Acta Paediatrica* **2017**, *106*, 918-925, doi:10.1111/apa.13829.

21. Andersen, G.S.; Girma, T.; Wells, J.C.K.; Kaestel, P.; Michaelsen, K.F.; Friis, H. Fat and Fat-Free Mass at Birth: Air Displacement Plethysmography Measurements on 350 Ethiopian Newborns. *Pediatric Research* **2011**, *70*, 501-506, doi:10.1203/PDR.0b013e31822d7470.

22. Carberry, A.E.; Colditz, P.B.; Lingwood, B.E. Body Composition From Birth to 4.5 Months in Infants Born to Non-Obese Women. *Pediatric Research* **2010**, *68*, 84-88, doi:10.1203/PDR.0b013e3181df5421.

23. Dong, P.; Feng, J.J.; Yan, D.Y.; Lyu, Y.J.; Xu, X. Children with cow's milk allergy following an elimination diet had normal growth but relatively low plasma leptin at age two. *Acta Paediatrica* **2018**, *107*, 1247-1252, doi:10.1111/apa.14283.

24. Baker, P.R.; Patinkin, Z.W.; Shapiro, A.L.B.; de la Houssaye, B.A.; Janssen, R.C.; Vanderlinden, L.A.; Dabelea, D.; Friedman, J.E. Altered gene expression and metabolism in fetal umbilical cord mesenchymal stem cells correspond with differences in 5-month-old infant adiposity gain. *Scientific Reports* **2017**, *7*, 10, doi:10.1038/s41598-017-17588-4.

25. Perng, W.; Ringham, B.M.; Glueck, D.H.; Sauder, K.A.; Starling, A.P.; Belfort, M.B.; Dabelea, D. An observational cohort study of weight- and length-derived anthropometric indicators with body composition at birth and 5 mo: the Healthy Start study. *American Journal of Clinical Nutrition* **2017**, *106*, 559-567, doi:10.3945/ajcn.116.149617.

26. Eriksson, B.; Lof, M.; Forsum, E. Body composition in full-term healthy infants measured with air displacement plethysmography at 1 and 12 weeks of age. *Acta Paediatrica* **2010**, *99*, 563-568, doi:10.1111/j.1651-2227.2009.01665.x.

27. Fields, D.A.; Krishnan, S.; Wisniewski, A.B. Sex Differences in Body Composition Early in Life. *Gender Medicine* **2009**, *6*, 369-375, doi:10.1016/j.genm.2009.07.003.

28. Fields, D.A.; Gilchrist, J.M.; Catalano, P.M.; Gianni, M.L.; Roggero, P.M.; Mosca, F. Longitudinal Body Composition Data in Exclusively Breast-Fed Infants: A Multicenter Study. *Obesity* **2011**, *19*, 1887-1891, doi:10.1038/oby.2011.11.

29. Fields, D.A.; Demerath, E.W.; Pietrobelli, A.; Chandler-Laney, P.C. Body Composition at 6 months of Life: Comparison Of Air Displacement Plethysmography and Dual-Energy X-Ray Absorptiometry. *Obesity* **2012**, *20*, 2302-2306, doi:10.1038/oby.2012.102.

30. Hull, H.R.; Dinger, M.K.; Knehans, A.W.; Thompson, D.M.; Fields, D.A. Impact of maternal body mass index on neonate birthweight and body composition. *American Journal of Obstetrics and Gynecology* **2008**, *198*, 6, doi:10.1016/j.ajog.2007.10.796.

31. Lingwood, B.E.; van Leeuwen, A.M.S.; Carberry, A.E.; Fitzgerald, E.C.; Callaway, L.K.; Colditz, P.B.; Ward, L.C. Prediction of fat-free mass and percentage of body fat in neonates using bioelectrical impedance analysis and anthropometric measures: validation against the PEA POD. *British Journal of Nutrition* **2012**, *107*, 1545-1552, doi:10.1017/s0007114511004624.

32. Olhager, E.; Tornqvist, C. Body composition in late preterm infants in the first 10days of life and at full term. *Acta Paediatrica* **2014**, *103*, 737-743, doi:10.1111/apa.12632.

33. Roggero, P.; Gianni, M.L.; Orsi, A.; Piemontese, P.; Amato, O.; Moioli, C.; Mosca, F. Neonatal period: body composition changes in breast-fed full-term newborns. *Neonatology* **2010**, *97*, 139-143, doi:10.1159/000239767.

34. Stanfield, K.M.; Wells, J.C.; Fewtrell, M.S.; Frost, C.; Leon, D.A. Differences in body composition between infants of South Asian and European ancestry: the London Mother and Baby Study. *International Journal of Epidemiology* **2012**, *41*, 1409-1418, doi:10.1093/ije/dys139.

35. Admassu, B.; Wells, J.C.K.; Girma, T.; Andersen, G.S.; Owino, V.; Belachew, T.; Michaelsen, K.F.; Abera, M.; Wibaek, R.; Friis, H., et al. Body composition at birth and height at 2 years: a prospective cohort study among children in Jimma, Ethiopia. *Pediatric Research* **2017**, *82*, 209-214, doi:10.1038/pr.2017.59.

36. Gianni, M.L.; Roggero, P.; Liotto, N.; Taroni, F.; Polimeni, A.; Morlacchi, L.; Piemontese, P.; Consonni, D.; Mosca, F. Body composition in late preterm infants according to percentile at birth. *Pediatric Research* **2016**, *79*, 710-715, doi:10.1038/pr.2015.273.

37. Kadakia, R.; Zheng, Y.; Zhang, Z.; Zhang, W.; Hou, L.; Josefson, J.L. Maternal pre-pregnancy BMI downregulates neonatal cord blood LEP methylation. *Pediatric Obesity* **2017**, *12*, 57-64, doi:10.1111/ijpo.12204.

38. Sauder, K.A.; Koeppen, H.J.; Shapiro, A.L.B.; Kalata, K.E.; Stamatoiu, A.V.; Ringham, B.M.; Glueck, D.H.; Norris, J.M.; Dabelea, D. Prenatal Vitamin D Intake, Cord Blood 25-Hydroxyvitamin D, and Offspring Body Composition: The Healthy Start Study. *Nutrients* **2017**, *9*, 14, doi:10.3390/nu9070790.

39. McKenzie, K.M.; Dissanayake, H.U.; McMullan, R.; Caterson, I.D.; Celermajer, D.S.; Gordon, A.; Hyett, J.; Meroni, A.; Phang, M.; Raynes-Greenow, C., et al. Quantity and Quality of Carbohydrate Intake during Pregnancy, Newborn Body Fatness and Cardiac Autonomic Control: Conferred Cardiovascular Risk? *Nutrients* **2017**, *9*, 12, doi:10.3390/nu9121375.

40. Abera, M.; Tesfaye, M.; Girma, T.; Hanlon, C.; Andersen, G.S.; Wells, J.C.; Admassu, B.; Wibaek, R.; Friis, H.; Kaestel, P. Relation between body composition at birth and child development at 2 years of age: a prospective cohort study among Ethiopian children. *European Journal of Clinical Nutrition* **2017**, *71*, 1411-1417, doi:10.1038/ejcn.2017.129.

41. Toro-Ramos, T.; Paley, C.; Wong, W.W.; Pi-Sunyer, F.X.; Yu, W.W.; Thornton, J.; Gallagher, D. Reliability of the EchoMRI Infants System for Water and Fat Measurements in Newborns. *Obesity* **2017**, *25*, 1577-1583, doi:10.1002/oby.21918.

42. Cauble, J.S.; Dewi, M.; Hull, H.R. Validity of anthropometric equations to estimate infant fat mass at birth and in early infancy. *Bmc Pediatrics* **2017**, *17*, 8, doi:10.1186/s12887-017-0844-6.

43. Chen, L.W.; Tint, M.T.; Fortier, M.V.; Aris, I.M.; Shek, L.P.C.; Tan, K.H.; Chan, S.Y.; Gluckman, P.D.; Chong, Y.S.; Godfrey, K.M., et al. Which anthropometric measures best reflect neonatal adiposity? *International Journal of Obesity* **2018**, *42*, 501-506, doi:10.1038/ijo.2017.250.

44. Chia, A.R.; Tint, M.T.; Han, C.Y.; Chen, L.W.; Colega, M.; Aris, I.M.; Chua, M.C.; Tan, K.H.; Yap, F.; Shek, L.P.C., et al. Adherence to a healthy eating index for pregnant women is associated with lower neonatal adiposity in a multiethnic Asian cohort: the Growing Up in Singapore Towards healthy Outcomes (GUSTO) Study. *American Journal of Clinical Nutrition* **2018**, *107*, 71-79, doi:10.1093/ajcn/nqx003.

45. Lemas, D.J.; Young, B.E.; Baker, P.R.; Tomczik, A.C.; Soderborg, T.K.; Hernandez, T.L.; de la Houssaye, B.A.; Robertson, C.E.; Rudolph, M.C.; Ir, D., et al. Alterations in human milk leptin and insulin are associated with early changes in the infant intestinal microbiome. *American Journal of Clinical Nutrition* **2016**, *103*, 1291-1300, doi:10.3945/ajcn.115.126375.

46. Breij, L.M.; Mulder, M.T.; van Vark-van der Zee, L.C.; Hokken-Koelega, A.C.S. Appetite-regulating hormones in early life and relationships with type of feeding and body composition in healthy term infants. *European Journal of Nutrition* **2017**, *56*, 1725-1732, doi:10.1007/s00394-016-1219-8.

47. Paley, C.; Hull, H.; Ji, Y.; Toro-Ramos, T.; Thornton, J.; Bauer, J.; Matthews, P.; Yu, A.; Navder, K.; Dorsey, K., et al. Body fat differences by self-reported race/ethnicity in healthy term newborns. *Pediatric Obesity* **2016**, *11*, 361-368, doi:10.1111/ijpo.12072.

48. Hawkes, C.P.; Zemel, B.S.; Kiely, M.; Irvine, A.D.; Kenny, L.C.; Hourihane, J.O.; Murray, D.M. Body Composition within the First 3 Months: Optimized Correction for Length and Correlation with BMI at 2 Years. *Hormone Research in Paediatrics* **2016**, *86*, 178-187, doi:10.1159/000448659.

49. McLeod, G.; Sherriff, J.; Hartmann, P.E.; Nathan, E.; Geddes, D.; Simmer, K. Comparing different methods of human breast milk fortification using measured v. assumed macronutrient composition to target reference growth: a randomised controlled trial. *British Journal of Nutrition* **2016**, *115*, 431-439, doi:10.1017/s0007114515004614.

50. Ramel, S.E.; Zhang, L.; Misra, S.; Anderson, C.G.; Demerath, E.W. Do anthropometric measures accurately reflect body composition in preterm infants? *Pediatric Obesity* **2017**, *12*, 72-77, doi:10.1111/ijpo.12181.

51. Gianni, M.L.; Consonni, D.; Liotto, N.; Roggero, P.; Morlacchi, L.; Piemontese, P.; Menis, C.; Mosca, F. Does Human Milk Modulate Body Composition in Late Preterm Infants at Term-Corrected Age? *Nutrients* **2016**, *8*, 10, doi:10.3390/nu8100664.

52. Kizirian, N.V.; Kong, Y.; Muirhead, R.; Brodie, S.; Garnett, S.P.; Petocz, P.; Sim, K.A.; Celermajer, D.S.; Louie, J.C.Y.; Markovic, T.P., et al. Effects of a low-glycemic index diet during pregnancy on offspring growth, body composition, and vascular health: a pilot randomized controlled trial. *American Journal of Clinical Nutrition* **2016**, *103*, 1073-1082, doi:10.3945/ajcn.115.123695.

53. Josefson, J.L.; Simons, H.; Zeiss, D.M.; Metzger, B.E. Excessive gestational weight gain in the first trimester among women with normal glucose tolerance and resulting neonatal adiposity. *Journal of Perinatology* **2016**, *36*, 1034-1038, doi:10.1038/jp.2016.145.

54. Scheurer, J.M.; Gray, H.L.; Demerath, E.W.; Rao, R.; Ramel, S.E. Diminished growth and lower adiposity in hyperglycemic very low birth weight neonates at 4 months corrected age. *Journal of Perinatology* **2016**, *36*, 145-150, doi:10.1038/jp.2015.154.

55. Sauder, K.A.; Starling, A.P.; Shapiro, A.L.; Kaar, J.L.; Ringham, B.M.; Glueck, D.H.; Dabelea, D. Exploring the association between maternal prenatal multivitamin use and early infant growth: The Healthy Start Study. *Pediatric Obesity* **2016**, *11*, 434-441, doi:10.1111/ijpo.12084.

56. Breij, L.M.; Kerkhof, G.F.; Rolfe, E.D.; Ong, K.K.; Abrahamse-Berkeveld, M.; Acton, D.; Hokken-Koelega, A.C.S. Longitudinal fat mass and visceral fat during the first 6months after birth in healthy infants: support for a critical window for adiposity in early life. *Pediatric Obesity* **2017**, *12*, 286-294, doi:10.1111/ijpo.12139.

57. Kizirian, N.V.; Markovic, T.P.; Muirhead, R.; Brodie, S.; Garnett, S.P.; Louie, J.C.Y.; Petocz, P.; Ross, G.P.; Brand-Miller, J.C. Macronutrient Balance and Dietary Glycemic Index in Pregnancy Predict Neonatal Body Composition. *Nutrients* **2016**, *8*, 13, doi:10.3390/nu8050270.

58. Crume, T.L.; Brinton, J.T.; Shapiro, A.; Kaar, J.; Glueck, D.H.; Siega-Riz, A.M.; Dabelea, D. Maternal dietary intake during pregnancy and offspring body composition: The Healthy Start Study. *Am J Obstet Gynecol* **2016**, *215*, 609.e601-609.e608, doi:10.1016/j.ajog.2016.06.035.

59. Shapiro, A.L.B.; Kaar, J.L.; Crume, T.L.; Starling, A.P.; Siega-Riz, A.M.; Ringham, B.M.; Glueck, D.H.; Norris, J.M.; Barbour, L.A.; Friedman, J.E., et al. Maternal diet quality in pregnancy and neonatal adiposity: the Healthy Start Study. *International Journal of Obesity* **2016**, *40*, 1056-1062, doi:10.1038/ijo.2016.79.

60. Tinius, R.A.; Cahill, A.G.; Strand, E.A.; Cade, W.T. Maternal inflammation during late pregnancy is lower in physically active compared with inactive obese women. *Applied Physiology Nutrition and Metabolism* **2016**, *41*, 191-198, doi:10.1139/apnm-2015-0316.

61. Barbour, L.A.; Hernandez, T.L.; Reynolds, R.M.; Reece, M.S.; Chartier-Logan, C.; Anderson, M.K.; Kelly, T.; Friedman, J.E.; Van Pelt, R.E. Striking differences in estimates of infant adiposity by new and old DXA software, PEAPOD and skin-folds at 2 weeks and 1 year of life. *Pediatric Obesity* **2016**, *11*, 264-271, doi:10.1111/ijpo.12055.

62. Starling, A.P.; Brinton, J.T.; Glueck, D.H.; Shapiro, A.L.; Harrod, C.S.; Lynch, A.M.; Siega-Riz, A.M.; Dabelea, D. Associations of maternal BMI and gestational weight gain with neonatal adiposity in the Healthy Start study. *American Journal of Clinical Nutrition* **2015**, *101*, 302-309, doi:10.3945/ajcn.114.094946.

63. Lemas, D.J.; Brinton, J.T.; Shapiro, A.L.B.; Glueck, D.H.; Friedman, J.E.; Dabelea, D. Associations of maternal weight status prior and during pregnancy with neonatal cardiometabolic markers at birth: the Healthy Start study. *International Journal of Obesity* **2015**, *39*, 1437-1442, doi:10.1038/ijo.2015.109.

64. Harrod, C.S.; Fingerlin, T.E.; Chasan-Taber, L.; Reynolds, R.M.; Glueck, D.H.; Dabelea, D. Exposure to Prenatal Smoking and Early-Life Body Composition: The Healthy Start Study. *Obesity* **2015**, *23*, 234-241, doi:10.1002/oby.20924.

65. Henriksson, P.; Lof, M.; Forsum, E. Parental fat-free mass is related to the fat-free mass of infants and maternal fat mass is related to the fat mass of infant girls. *Acta Paediatrica* **2015**, *104*, 491-497, doi:10.1111/apa.12939.

66. Crume, T.L.; Shapiro, A.L.; Brinton, J.T.; Glueck, D.H.; Martinez, M.; Kohn, M.; Harrod, C.; Friedman, J.E.; Dabelea, D. Maternal Fuels and Metabolic Measures During Pregnancy and Neonatal Body Composition: The Healthy Start Study. *Journal of Clinical Endocrinology & Metabolism* **2015**, *100*, 1672-1680, doi:10.1210/jc.2014-2949.

67. Roggero, P.; Gianni, M.L.; Forzenigo, L.; Tondolo, T.; Taroni, F.; Liotto, N.; Piemontese, P.; Biondetti, P.; Mosca, F. No relative increase in intra-abdominal adipose tissue in healthy unstressed preterm infants at term. *Neonatology* **2015**, *107*, 14-19, doi:10.1159/000364855.

68. Wibaek, R.; Kaestel, P.; Skov, S.R.; Christensen, D.L.; Girma, T.; Wells, J.C.K.; Friis, H.; Andersen, G.S. Calibration of bioelectrical impedance analysis for body composition assessment in Ethiopian infants using air-displacement plethysmography. *European Journal of Clinical Nutrition* **2015**, *69*, 1099-1104, doi:10.1038/ejcn.2015.51.

69. Breij, L.M.; Steegers-Theunissen, R.P.M.; Briceno, D.; Hokken-Koelega, A.C.S. Maternal and Fetal Determinants of Neonatal Body Composition. *Hormone Research in Paediatrics* **2015**, *84*, 388-395, doi:10.1159/000441298.

70. De Cunto, A.; Paviotti, G.; Travan, L.; Bua, J.; Cont, G.; Demarini, S. Impact of Surgery for Neonatal Gastrointestinal Diseases on Weight and Fat Mass. *Journal of Pediatrics* **2015**, *167*, 568-571, doi:10.1016/j.jpeds.2015.06.013.

71. Henriksson, P.; Eriksson, B.; Forsum, E.; Lof, M. Gestational weight gain according to Institute of Medicine recommendations in relation to infant size and body composition. *Pediatric Obesity* **2015**, *10*, 388-394, doi:10.1111/ijpo.276.

72. McLeod, G.; Simmer, K.; Sherriff, J.; Nathan, E.; Geddes, D.; Hartmann, P. Feasibility study: Assessing the influence of macronutrient intakes on preterm body composition, using air displacement plethysmography. *Journal of Paediatrics and Child Health* **2015**, *51*, 862-869, doi:10.1111/jpc.12893.

73. Donnelley, E.L.; Raynes-Greenow, C.H.; Turner, R.M.; Carberry, A.E.; Jeffery, H.E. Antenatal predictors and body composition of large-for-gestational-age newborns: perinatal health outcomes. *Journal of Perinatology* **2014**, *34*, 698-704, doi:10.1038/jp.2014.90.

74. Gianni, M.L.; Roggero, P.; Piemontese, P.; Morlacchi, L.; Bracco, B.; Taroni, F.; Garavaglia, E.; Mosca, F. Boys who are born preterm show a relative lack of fat-free mass at 5 years of age compared to their peers. *Acta Paediatrica* **2015**, *104*, E119-E123, doi:10.1111/apa.12856.

75. Simon, L.; Frondas-Chauty, A.; Senterre, T.; Flamant, C.; Darmaun, D.; Roze, J.C. Determinants of body composition in preterm infants at the time of hospital discharge. *American Journal of Clinical Nutrition* **2014**, *100*, 98-104, doi:10.3945/ajcn.113.080945.

76. Gianni, M.L.; Roggero, P.; Morlacchi, L.; Garavaglia, E.; Piemontese, P.; Mosca, F. Formula-fed infants have significantly higher fat-free mass content in their bodies than breastfed babies. *Acta Paediatrica* **2014**, *103*, e277-e281, doi:10.1111/apa.12643.

77. Josefson, J.L.; Zeiss, D.M.; Rademaker, A.W.; Metzger, B.E. Maternal Leptin Predicts Adiposity of the Neonate. *Hormone Research in Paediatrics* **2014**, *81*, 13-19, doi:10.1159/000355387.

78. Hull, H.R.; Thornton, J.; Paley, C.; Navder, K.; Gallagher, D. Maternal obesity influences the relationship between location of neonate fat mass and total fat mass. *Pediatric Obesity* **2015**, *10*, 245-251, doi:10.1111/ijpo.257.

79. Harrod, C.S.; Chasan-Taber, L.; Reynolds, R.M.; Fingerlin, T.E.; Glueck, D.H.; Brinton, J.T.; Dabelea, D. Physical Activity in Pregnancy and Neonatal Body Composition The Healthy Start Study. *Obstetrics and Gynecology* **2014**, *124*, 257-264, doi:10.1097/aog.0000000000000373.

80. Harrod, C.S.; Reynolds, R.M.; Chasan-Taber, L.; Fingerlin, T.E.; Glueck, D.H.; Brinton, J.T.; Dabelea, D. Quantity and Timing of Maternal Prenatal Smoking on Neonatal Body Composition: The Healthy Start Study. *Journal of Pediatrics* **2014**, *165*, 707-712, doi:10.1016/j.jpeds.2014.06.031.

81. Pereira-da-Silva, L.; Cabo, C.; Moreira, A.C.; Virella, D.; Guerra, T.; Camoes, T.; Silva, A.R.; Neves, R.; Ferreira, G.C. The Adjusted Effect of Maternal Body Mass Index, Energy and Macronutrient Intakes during Pregnancy, and Gestational Weight Gain on Body Composition of Full-Term Neonates. *American Journal of Perinatology* **2014**, *31*, 875-881, doi:10.1055/s-0033-1363502.

82. Henriksson, P.; Lof, M.; Soderkvist, P.; Forsum, E. Variation in the fat mass and obesity-related (FTO) genotype is not associated with body fatness in infants, but possibly with their length. *Pediatric Obesity* **2014**, *9*, E112-E115, doi:10.1111/ijpo.231.

83. Andersen, G.S.; Girma, T.; Wells, J.C.K.; Kaestel, P.; Leventi, M.; Hother, A.L.; Michaelsen, K.F.; Friis, H. Body composition from birth to 6 mo of age in Ethiopian infants: reference data obtained by air-displacement plethysmography. *American Journal of Clinical Nutrition* **2013**, *98*, 885-894, doi:10.3945/ajcn.113.063032.

84. Au, C.P.; Raynes-Greenow, C.H.; Turner, R.M.; Carberry, A.E.; Jeffery, H.E. Body composition is normal in term infants born to mothers with well-controlled gestational diabetes mellitus. *Diabetes Care* **2013**, *36*, 562-564, doi:10.2337/dc12-1557.

85. Carberry, A.E.; Raynes-Greenow, C.H.; Turner, R.M.; Jeffery, H.E. Customized Versus Population-Based Birth Weight Charts for the Detection of Neonatal Growth and Perinatal Morbidity in a Cross-Sectional Study of Term Neonates. *American Journal of Epidemiology* **2013**, *178*, 1301-1308, doi:10.1093/aje/kwt176.

86. Josefson, J.L.; Hoffmann, J.A.; Metzger, B.E. Excessive weight gain in women with a normal pre-pregnancy BMI is associated with increased neonatal adiposity. *Pediatric Obesity* **2013**, *8*, e33-e36, doi:10.1111/j.2047-6310.2012.00132.x.

87. Pfister, K.M.; Gray, H.L.; Miller, N.C.; Demerath, E.W.; Georgieff, M.K.; Ramel, S.E. Exploratory study of the relationship of fat-free mass to speed of brain processing in preterm infants. *Pediatric Research* **2013**, *74*, 576-583, doi:10.1038/pr.2013.138.

88. Carberry, A.E.; Raynes-Greenow, C.H.; Turner, R.M.; Askie, L.M.; Jeffery, H.E. Is body fat percentage a better measure of undernutrition in newborns than birth weight percentiles? *Pediatric Research* **2013**, *74*, 730-736, doi:10.1038/pr.2013.156.

89. Josefson, J.L.; Feinglass, J.; Rademaker, A.W.; Metzger, B.E.; Zeiss, D.M.; Price, H.E.; Langman, C.B. Maternal Obesity and Vitamin D Sufficiency Are Associated with Cord Blood Vitamin D Insufficiency. *Journal of Clinical Endocrinology & Metabolism* **2013**, *98*, 114-119, doi:10.1210/jc.2012-2882.

90. Paviotti, G.; De Cunto, A.; Travan, L.; Bua, J.; Cont, G.; Demarini, S. Longitudinal Growth and Body Composition of Twins versus Singletons in the First Month of Life. *Scientific World Journal* **2013**, 10.1155/2013/108189, 3, doi:10.1155/2013/108189.

91. Meyers, J.M.; Greecher, C.P.; Shaffer, M.L.; Shenberger, J.S. Potential influence of total parenteral nutrition on body composition at discharge in preterm infants. *Journal of Maternal-Fetal & Neonatal Medicine* **2013**, *26*, 1548-1553, doi:10.3109/14767058.2013.793663.

92. Deierlein, A.L.; Thornton, J.; Hull, H.; Paley, C.; Gallagher, D. An anthropometric model to estimate neonatal fat mass using air displacement plethysmography. *Nutrition & Metabolism* **2012**, *9*, 5, doi:10.1186/1743-7075-9-21.

93. Eriksson, B.; Henriksson, H.; Lof, M.; Hannestad, U.; Forsum, E. Body-composition development during early childhood and energy expenditure in response to physical activity in 1.5-y-old children. *American Journal of Clinical Nutrition* **2012**, *96*, 567-573, doi:10.3945/ajcn.111.022020.

94. Hull, H.R.; Thornton, J.C.; Ji, Y.; Paley, C.; Rosenn, B.; Mathews, P.; Navder, K.; Yu, A.; Dorsey, K.; Gallagher, D. Higher infant body fat with excessive gestational weight gain in overweight women. *American Journal of Obstetrics and Gynecology* **2011**, *205*, 7, doi:10.1016/j.ajog.2011.04.004.

95. Taroni, F.; Forzenigo, L.; Tondolo, T.; Liotto, N.; Bracco, B.; Garavaglia, E.; Garbarino, F.; Biondetti, P.; Mosca, F. [Intra-abdominal adiposity in preterm infants: an explorative study]. *Pediatr Med Chir* **2012**, *34*, 283-286, doi:10.4081/pmc.2012.52.

96. Andres, A.; Shankar, K.; Badger, T.M. Body Fat Mass of Exclusively Breastfed Infants Born to Overweight Mothers. *Journal of the Academy of Nutrition and Dietetics* **2012**, *112*, 991-995, doi:10.1016/j.jand.2012.03.031.

97. Roggero, P.; Gianni, M.L.; Orsi, A.; Piemontese, P.; Amato, O.; Liotto, N.; Morlacchi, L.; Taroni, F.; Fields, D.A.; Catalano, P.M., et al. Quality of Growth in Exclusively Breast-Fed Infants in the First Six Months of Life: An Italian Study. *Pediatric Research* **2010**, *68*, 542-544, doi:10.1203/PDR.0b013e3181f85a20.

98. Law, T.L.; Korte, J.E.; Katikaneni, L.D.; Wagner, C.L.; Ebeling, M.D.; Newman, R.B. Ultrasound assessment of intrauterine growth restriction: relationship to neonatal body composition. *American Journal of Obstetrics and Gynecology* **2011**, *205*, 6, doi:10.1016/j.ajog.2011.06.027.

99. Algotar, A.; Shaikhkhalil, A.K.; Siler-Wurst, K.; Sitaram, S.; Gulati, I.; Jadcherla, S.R. Unique Patterns of Body Composition and Anthropometric Measurements During Maturation in Neonatal Intensive Care Unit Neonates: Opportunities for Modifying Nutritional Therapy and Influencing Clinical Outcomes. *Journal of Parenteral and Enteral Nutrition* **2018**, *42*, 231-238, doi:10.1002/jpen.1012.

100. Piemontese, P.; Liotto, N.; Mallardi, D.; Roggero, P.; Puricelli, V.; Gianni, M.L.; Morniroli, D.; Tabasso, C.; Perrone, M.; Menis, C., et al. The Effect of Human Milk on Modulating the Quality of Growth in Preterm Infants. *Frontiers in Pediatrics* **2018**, *6*, 7, doi:10.3389/fped.2018.00291.

101. Moyer-Mileur, L.J.; Slater, H.; Thomson, J.A.; Mihalopoulos, N.; Byrne, J.; Varner, M.W. Newborn Adiposity Measured by Plethysmography Is Not Predicted by Late Gestation Two-Dimensional Ultrasound Measures of Fetal Growth. *Journal of Nutrition* **2009**, *139*, 1772-1778, doi:10.3945/jn.109.109058.

102. Anderson, A.K.; McDougald, D.M.; Steiner-Asiedu, M. Dietary trans fatty acid intake and maternal and infant adiposity. *European Journal of Clinical Nutrition* **2010**, *64*, 1308-1315, doi:10.1038/ejcn.2010.166.

103. Gianni, M.L.; Roggero, P.; Liotto, N.; Amato, O.; Piemontese, P.; Morniroli, D.; Bracco, B.; Mosca, F. Postnatal catch-up fat after late preterm birth. *Pediatric Research* **2012**, *72*, 637-640, doi:10.1038/pr.2012.128.

104. Lee, W.; Riggs, T.; Koo, W.; Deter, R.L.; Yeo, L.; Romero, R. The relationship of newborn adiposity to fetal growth outcome based on birth weight or the modified neonatal growth assessment score. *Journal of Maternal-Fetal & Neonatal Medicine* **2012**, *25*, 1933-1940, doi:10.3109/14767058.2012.683084.

105. Aris, I.M.; Soh, S.E.; Tint, M.T.; Liang, S.; Chinnadurai, A.; Saw, S.M.; Kwek, K.; Godfrey, K.M.; Gluckman, P.D.; Chong, Y.S., et al. Body fat in Singaporean infants: development of body fat prediction equations in Asian newborns. *European Journal of Clinical Nutrition* **2013**, *67*, 922-927, doi:10.1038/ejcn.2013.69.

106. Markovic, T.P.; Muirhead, R.; Overs, S.; Ross, G.P.; Louie, J.C.Y.; Kizirian, N.; Denyer, G.; Petocz, P.; Hyett, J.; Brand-Miller, J.C. Randomized Controlled Trial Investigating the Effects of a Low-Glycemic Index Diet on Pregnancy Outcomes in Women at High Risk of Gestational Diabetes Mellitus: The GI Baby 3 Study. *Diabetes Care* **2016**, *39*, 31-38, doi:10.2337/dc15-0572.

107. Tinius, R.A.; Cahill, A.G.; Strand, E.A.; Cade, W.T. Altered maternal lipid metabolism is associated with higher inflammation in obese women during late pregnancy. *Integrative obesity and diabetes* **2015**, *2*, 168-175.

108. Kadakia, R.; Ma, M.; Josefson, J.L. Neonatal adiposity increases with rising cord blood IGF-1 levels. *Clinical Endocrinology* **2016**, *85*, 70-75, doi:10.1111/cen.13057.

109. Hawkes, C.P.; Hourihane, J.O.; Kenny, L.C.; Irvine, A.D.; Kiely, M.; Murray, D.M. Gender- and Gestational Age-Specific Body Fat Percentage at Birth. *Pediatrics* **2011**, *128*, E645-E651, doi:10.1542/peds.2010-3856.

110. Tint, M.T.; Ward, L.C.; Soh, S.E.; Aris, I.M.; Chinnadurai, A.; Saw, S.M.; Gluckman, P.D.; Godfrey, K.M.; Chong, Y.S.; Kramer, M.S., et al. Estimation of fat-free mass in Asian neonates using bioelectrical impedance analysis. *British Journal of Nutrition* **2016**, *115*, 1033-1042, doi:10.1017/s0007114515005486.
