# Supplementary material for: Fat and Fat-Free Mass of Preterm and Term Infants from Birth to Six Months: A Review of Current Evidence
Source: Nutrients. 2020 Jan 21;12(2):288. doi: 10.3390/nu12020288 (PMC7070317; doi:10.3390/nu12020288)
Supplement: Supplementary file 1 [file nutrients-12-00288-s001.zip › Suppl-2 DXA 2020-01-08.docx]

**Table S2:** Overview of reviewed articles with body composition measurements using dual energy X-ray absorptiometry (DXA) in preterm and term infants

AGA: appropriate for gestational age, BF: breastfed, CLD: chronic lung disease, CMF: cow milk formula, FGR: fetal growth restriction, FHM: fortified human milk, HM: human milk, LGA. large for gestational age, MF. mixed feeding, N: normal, PDF: postdischarge formula, PMA: postmenstrual age PTF: preterm formula, SF: soy formula, SGA: small for gestational age, TF: term formula, VLBW: very low birth weight

| **Name** | **Year of study** | **Country** | **Type of study** | **Number, gestational age, birth weight** | **Age at study (weeks PMA)** | **Info** |
| --- | --- | --- | --- | --- | --- | --- |
| Griffin 2009 [1] | unknown | UK | longitudinal | n=149, 31 wks, 1406g | 37 wks |  |
| Hammami 2003 [2] | unknown |  |  | n=76, 39.5 wks, 3354g | 39.9 wks |  |
| Cooke 2010 [3] | unknown | unknown | longitudinal | n= 56, 31.3 wks, 1414g | 40, 43, 55, 83 wks | Preterm formula |
|  |  |  |  | n=57, 30.9 wks, 1402g | 40, 42.9, 54.9, 82.9 wks | Term formula |
|  |  |  |  | n=26, 30.7 wks, 1349g | 40, 42.7, 54.7, 82.7 wks | Crossover |
|  |  |  |  | n=25, 30.7 wks, 1377g | 40, 42.7, 54.7, 82.7 wks | BF |
| Lapilonne 1997 [4] | unknown | France | cross-sectional | n=6, 32.5 wks, 2125g | 32.6 wks | AGA |
|  |  |  |  | n=22, 34.5 wks, 2371g | 34.6 wks | AGA |
|  |  |  |  | n=7, 36.5 wks, 2701g | 36.6 wks | AGA |
|  |  |  |  | n=9, 36.5 wks, 1992g | 36.5 wks | SGA |
|  |  |  |  | n=19, 38.5 wks, 2992g | 38.6 wks | AGA |
|  |  |  |  | n=7, 38.5 wks, 2286g | 38.6 wks | SGA |
|  |  |  |  | n=16, 40.5 wks, 3521g | 40.6 wks | AGA |
|  |  |  |  | n=4, 40.5 wks, 2100g | 40.6 wks | SGA |
| Amesz 2010 [5] | 2003-2006 | Netherlands | longitudinal | n=43, 30.7 wks, 1345g | 40.7, 64 wks | PDF |
|  |  |  |  | n=34, 30.9 wks, 1377g | 40.7, 64 wks | TF |
|  |  |  |  | n=6, 30 wks, 1291g | 40.7, 64 wks | HM |
| De Curtis 2002 [6] | unknown | Belgium | longitudinal | n=16, 30 wks, 1294g | 36.4, 45.1 wks | Postdischarge formula |
|  |  |  |  | n=17, 30 wks, 1261g | 36.4, 54.4 wks | Standard |
| van Poelje 2014 [7] | unknown | Netherlands | longitudinal | n=139, 1340 g | 40, 60 wks | MF |
| Ahmad 2010 [8] | unknown | USA | cross-sectional | n=20, 25.9 wks, 864.8g | 38.6 wks |  |
|  |  |  |  | n=20, 30.8 wks, 1609g | 35.3 wks |  |
|  |  |  |  | n=17, 34.2 wks, 2225g | 35.6 wks |  |
|  |  |  |  | n=39, 39.4 wks, 3519g | 40.1 wks |  |
| Bolt 2002 [9] | unknown | unknown | longitudinal | n=11, 28.5 wks, 1005g | 40, 52 wks | CLD, MF |
|  |  |  |  | n=18, 29.3 wks, 1113g | 40, 52 wks | no CLD, MF |
| Pieltain 2001 [10] | unknown | unknown | longitudinal | n=20, 31 wks, 1298g | 34, 37.3 wks | FHM |
|  |  |  |  | n=34, 30 wks, 1269g | 33.1, 36.7 wks | PTF |
| de Pipaon 2017 [11] | 2004-2006 | Spain | longitudinal | n=82, 29 wks, 1060g | 64 wks | VLBW |
| Vignochi 2008 [12] | 2006 | Brasilia | longitudinal | n=15, 30.8 wks, 1326g | 34, 37.5 wks | Physical therapy |
|  |  |  |  | n=14, 30.7 wks, 1341g | 33.7, 37.1 wks | Control |
| Mehta 1998 [13] |  | USA | longitudinal | n=71, 40 wks, | 52, 64 wks | Early introduction of solid food |
|  |  |  |  | n=76, 40 wks | 52, 64 wks | Late introduction of solid food |
|  |  |  |  | n=76, 40 wks | 52, 64 wks | Commercially prepared solid food |
|  |  |  |  | n=71, 40 wks | 52, 64 wks | Parents´ choice of solid food |
| Atkinson 2000 [14] | 1991-1998 | Canada | cross-sectional | n=18, 26 wks, 845g | 40 wks | AGA EEF |
|  |  |  |  | n=34, 30 wks, 1324g | 40 wks | AGA, standard term formula |
|  |  |  |  | n=37, 30 wks, 1260g | 40 wks | AGA, nutrient enriched formula |
|  |  |  |  | n=27, 29 wks, 1187g | 40 wks | Mother´s milk |
|  |  |  |  | n=35, 33 wks, 1334g | 40 wks | SGA |
|  |  |  |  | n=46, 40 wks, 3376g | 40,1 wks | Term reference |
| Schmelzle 2002 [15] | 1998-1999 | Germany | longitudinal | n=68, 38.3 wks, | 39.2, 46.8, 54.8 wks | MF |
| Koo 2004 [16] |  | USA | cross-sectional | n=74, 35.9 wks, 2454g | 36.3 wks | AGA |
|  |  |  |  | n=16, 36.5 wks, 1971g | 36.9 wks | SGA |
|  |  |  |  | n=30, 39.7 wks, 4439g | 40.1 wks | LGA |
| Schmelzle 2007 [17] | unknown | Germany | cross-sectional | n=35, 33.9 wks, 2120g | 34 wks | Preterm |
|  |  |  |  | n=124, 39.7 wks, 3420g | 40.3 wks | Term, MF |
|  |  |  |  | n=26, 38.2 wks, 2320g | 38,9 wks | SGA, MF |
|  |  |  |  | n=118, 38.3 wks, 3150g | 39 wks | AGA, MF |
|  |  |  |  | n=15, 39.2 wks, 4430g | 39.9 wks | LGA, MF |
| Verkauskiene 2007 [18] | 2003-2005 | France | cross-sectional | n=45, 38.4 wks, 2509g | 38.8 wks | SGA-FGR |
|  |  |  |  | n=44, 38.7 wks, 2520g | 39.1 wks | SGA-N |
|  |  |  |  | n=60, 38.9 wks, 2986g | 39.3 wks | AGA-FGR |
|  |  |  |  | n=99, 39.3 wks, 3372g | 39.7 wks | AGA-N |
| Petry 2010 [19] | 2006-2008 | Spain | cross-sectional | n=193, 39.3 wks, 3100g | 41.3 wks | T/T mutation |
|  |  |  |  | n=104, 39.1 wks, 3100g | 41.2 wks | C/T mutation |
|  |  |  |  | n=15, 39.8 wks, 3000g | 41.8 wks | C/C mutation |
| Holroyd 2012 [20] |  | UK | cross-sectional | n=474, 40.1 wks, 3590g | 41 wks |  |
|  |  |  |  | n=440, 40.3 wks, 3490g | 41.2 wks |  |
| Andres 2013 [21] |  | USA | longitudinal | n=37, 39.3 wks, 3500g | 51.3, 63.3 wks | BF |
|  |  |  |  | n=19, 39.3 wks, 3500g | 51.3, 63.3 wks | CMF, MF |
|  |  |  |  | n=10, 39.3 wks, 3500g | 51.3, 63.3 wks | SF, MF |
| Friis 2013 [22] | 2001-2008 | Norway | cross-sectional | n=207, 40.1 wks, 3592g | 40.4 wks |  |
| Carlsen 2014 [23] | 2010-1012 | Denmark | cross-sectional | n=80, 39.9 wks, 3484g | 40.1 wks | normal weight mothers |
|  |  |  |  | n=231, 40 wks, 3666g | 40.2 wks | obese mothers |
| Alderete 2015 [24] |  | USA | longitudinal | n=32, 39.6 wks, 3560g | 45.1, 63.5 wks | BF |
| Carlsen 2015 [25] |  | Denmark | cross-sectional | n=130, 40.1 wks, 3728g | 40.3 wks | obese mothers (pre-pregnancy BMI 34.4) |
|  |  |  |  | n=44, 40.1 wks, 3572g | 40.3 wks | normal weight mothers (pre-pregnancy BMI: 22.2) |
| Boghossian 2019 [26] | 1992-1995 | USA | longitudinal | n=252, 39.3 wks, 3070g | 39.5 wks |  |
| Hellmuth 2019 [27] |  | USA | cross-sectional | n=121, 39.4 wks, 3430g | 42.4 wks |  |
| Bisson 2016 [28] | 2012-2015 | Canada | cross-sectional | n=104, 39.7 wks, 3448g | 41.5 wks |  |

**References**:

1. Cooke, R.J.; Griffin, I. Altered body composition in preterm infants at hospital discharge. *Acta Paediatrica* **2009**, *98*, 1269-1273, doi:10.1111/j.1651-2227.2009.01354.x.

2. Hammami, M.; Koo, W.W.K.; Hockman, E.M. Body composition of neonates from fan beam dual energy X-ray absorptiometry measurement. *Journal of Parenteral and Enteral Nutrition* **2003**, *27*, 423-426, doi:10.1177/0148607103027006423.

3. Cooke, R.J.; Griffin, I.J.; McCormick, K. Adiposity Is Not Altered in Preterm Infants Fed With a Nutrient-Enriched Formula After Hospital Discharge. *Pediatric Research* **2010**, *67*, 660-664, doi:10.1203/PDR.0b013e3181da8d01.

4. Lapillonne, A.; Braillon, P.; Claris, O.; Chatelain, P.G.; Delmas, P.D.; Salle, B.L. Body composition in appropriate and in small for gestational age infants. *Acta Paediatr* **1997**, *86*, 196-200, doi:10.1111/j.1651-2227.1997.tb08868.x.

5. Amesz, E.M.; Schaafsma, A.; Cranendonk, A.; Lafeber, H.N. Optimal Growth and Lower Fat Mass in Preterm Infants Fed a Protein-enriched Postdischarge Formula. *Journal of Pediatric Gastroenterology and Nutrition* **2010**, *50*, 200-207, doi:10.1097/MPG.0b013e3181a8150d.

6. De Curtis, M.; Pieltain, C.; Rigo, J. Body composition in preterm infants fed standard term or enriched formula after hospital discharge. *European Journal of Nutrition* **2002**, *41*, 177-182, doi:10.1007/500394-002-0374-2.

7. van Poelje, M.W.; van de Lagemaat, M.; Lafeber, H.N.; Van Weissenbruch, M.M.; Rotteveel, J. Relationship between Fat Mass Measured by Dual-Energy X-Ray Absorptiometry and Leptin in Preterm Infants between Term Age and 6 Months' Corrected Age. *Hormone Research in Paediatrics* **2014**, *82*, 405-410, doi:10.1159/000369393.

8. Ahmad, I.; Nemet, D.; Eliakim, A.; Koeppel, R.; Grochow, D.; Coussens, M.; Gallitto, S.; Rich, J.; Pontello, A.; Leu, S.Y., et al. Body Composition and Its Components in Preterm and Term Newborns: A Cross-Sectional, Multimodal Investigation. *American Journal of Human Biology* **2010**, *22*, 69-75, doi:10.1002/ajhb.20955.

9. Bolt, R.J.; van Weissenbruch, M.M.; Roos, J.C.; Delemaare-van de Waal, H.A.; Cranendonk, A.; Lafeber, H.N. Body composition in infants with chronic lung disease after treatment with dexamethasone. *Acta Paediatrica* **2002**, *91*, 815-821, doi:10.1080/08035250213220.

10. Pieltain, C.; De Curtis, M.; Gerard, P.; Rigo, J. Weight gain composition in preterm infants with dual energy X-ray absorptiometry. *Pediatric Research* **2001**, *49*, 120-124, doi:10.1203/00006450-200101000-00023.

11. de Pipaon, M.S.; Dorronsoro, I.; Alvarez-Cuervo, L.; Butte, N.F.; Madero, R.; Barrios, V.; Coya, J.; Martinez-Biarge, M.; Martos-Moreno, G.A.; Fewtrell, M.S., et al. The impact of intrauterine and extrauterine weight gain in premature infants on later body composition. *Pediatric Research* **2017**, *82*, 658-664, doi:10.1038/pr.2017.123.

12. Vignochi, C.M.; Miura, E.; Canani, L.H. Effects of motor physical therapy on bone mineralization in premature infants: a randomized controlled study. *Journal of Perinatology* **2008**, *28*, 624-631, doi:10.1038/jp.2008.60.

13. Mehta, K.C.; Specker, B.L.; Bartholmey, S.; Giddens, J.; Ho, M.L. Trial on timing of introduction to solids and food type on infant growth. *Pediatrics* **1998**, *102*, 569-573, doi:10.1542/peds.102.3.569.

14. Atkinson, S.A.; Randall-Simpson, J. Factors influencing body composition of premature infants at term-adjusted age. *Ann.NY Acad.Sci.* **2000**, *904*, 393-399.

15. Schmelzle, H.R.; Fusch, C. Body fat in neonates and young infants: validation of skinfold thickness versus dual-energy X-ray absorptiometry. *American Journal of Clinical Nutrition* **2002**, *76*, 1096-1100.

16. Koo, W.W.K.; Walters, J.C.; Hockman, E.M. Body composition in neonates: Relationship between measured and derived anthropometry with dual-energy x-ray absorptiometry measurements. *Pediatric Research* **2004**, *56*, 694-700, doi:10.1203/01.pdr.0000142587.59238.bd.

17. Schmelzle, H.R.; Quang, D.N.; Fusch, G.; Fusch, C. Birth weight categorization according to gestational age does not reflect percentage body fat in term and preterm newborns. *Eur J Pediatr* **2007**, *166*, 161-167, doi:10.1007/s00431-006-0209-x.

18. Verkauskiene, R.; Beltrand, J.; Claris, O.; Chevenne, D.; Deghmoun, S.; Dorgeret, S.; Alison, M.; Gaucherand, P.; Sibony, O.; Levy-Marchal, C. Impact of fetal growth restriction on body composition and hormonal status at birth in infants of small and appropriate weight for gestational age. *European Journal of Endocrinology* **2007**, *157*, 605-612, doi:10.1530/eje-07-0286.

19. Petry, C.J.; Lopez-Bermejo, A.; Diaz, M.; Sebastiani, G.; Ong, K.K.; de Zegher, F.; Dunger, D.B.; Ibanez, L. Association between a Common Variant near MC4R and Change in Body Mass Index Develops by Two Weeks of Age. *Hormone Research in Paediatrics* **2010**, *73*, 275-280, doi:10.1159/000284392.

20. Holroyd, C.R.; Harvey, N.C.; Crozier, S.R.; Winder, N.R.; Mahon, P.A.; Ntani, G.; Godfrey, K.M.; Inskip, H.M.; Cooper, C.; Grp, S.W.S.S. Placental size at 19 weeks predicts offspring bone mass at birth: Findings from the Southampton Women's Survey. *Placenta* **2012**, *33*, 623-629, doi:10.1016/j.placenta.2012.04.007.

21. Andres, A.; Casey, P.H.; Cleves, M.A.; Badger, T.M. Body Fat and Bone Mineral Content of Infants Fed Breast Milk, Cow's Milk Formula, or Soy Formula during the First Year of Life. *Journal of Pediatrics* **2013**, *163*, 49-54, doi:10.1016/j.jpeds.2012.12.067.

22. Friis, C.M.; Qvigstad, E.; Roland, M.C.P.; Godang, K.; Voldner, N.; Bollerslev, J.; Henriksen, T. Newborn Body Fat: Associations with Maternal Metabolic State and Placental Size. *Plos One* **2013**, *8*, 7, doi:10.1371/journal.pone.0057467.

23. Carlsen, E.M.; Renault, K.M.; Norgaard, K.; Nilas, L.; Jensen, J.E.B.; Hyldstrup, L.; Michaelsen, K.F.; Cortes, D.; Pryds, O. Newborn regional body composition is influenced by maternal obesity, gestational weight gain and the birthweight standard score. *Acta Paediatrica* **2014**, *103*, 939-945, doi:10.1111/apa.12713.

24. Alderete, T.L.; Autran, C.; Brekke, B.E.; Knight, R.; Bode, L.; Goran, M.I.; Fields, D.A. Associations between human milk oligosaccharides and infant body composition in the first 6 mo of life. *American Journal of Clinical Nutrition* **2015**, *102*, 1381-1388, doi:10.3945/ajcn.115.115451.

25. Carlsen, E.M.; Renault, K.M.; Jensen, R.B.; Norgaard, K.; Jensen, J.E.B.; Nilas, L.; Cortes, D.; Michaelsen, K.F.; Pryds, O. The Association between Newborn Regional Body Composition and Cord Blood Concentrations of C-Peptide and Insulin-Like Growth Factor I. *Plos One* **2015**, *10*, 14, doi:10.1371/journal.pone.0121350.

26. Boghossian, N.S.; Koo, W.; Liu, A.; Mumford, S.L.; Tsai, M.Y.; Yeung, E.H. Longitudinal measures of maternal vitamin D and neonatal body composition. *European Journal of Clinical Nutrition* **2019**, *73*, 424-431, doi:10.1038/s41430-018-0212-0.

27. Hellmuth, C.; Lindsay, K.L.; Uhl, O.; Buss, C.; Wadhwa, P.D.; Koletzko, B.; Entringer, S. Maternal Metabolomic Profile and Fetal Programming of Offspring Adiposity: Identification of Potentially Protective Lipid Metabolites. *Molecular Nutrition & Food Research* **2019**, *63*, 11, doi:10.1002/mnfr.201700889.

28. Bisson, M.; Tremblay, F.; St-Onge, O.; Robitaille, J.; Pronovost, E.; Simonyan, D.; Marc, I. Influence of maternal physical activity on infant's body composition. *Pediatric Obesity* **2017**, *12*, 38-46, doi:10.1111/ijpo.12174.
