# Supplementary material for: Fat and Fat-Free Mass of Preterm and Term Infants from Birth to Six Months: A Review of Current Evidence
Source: Nutrients. 2020 Jan 21;12(2):288. doi: 10.3390/nu12020288 (PMC7070317; doi:10.3390/nu12020288)
Supplement: Supplementary file 1 [file nutrients-12-00288-s001.zip › Suppl-3 2020-01-08.docx]

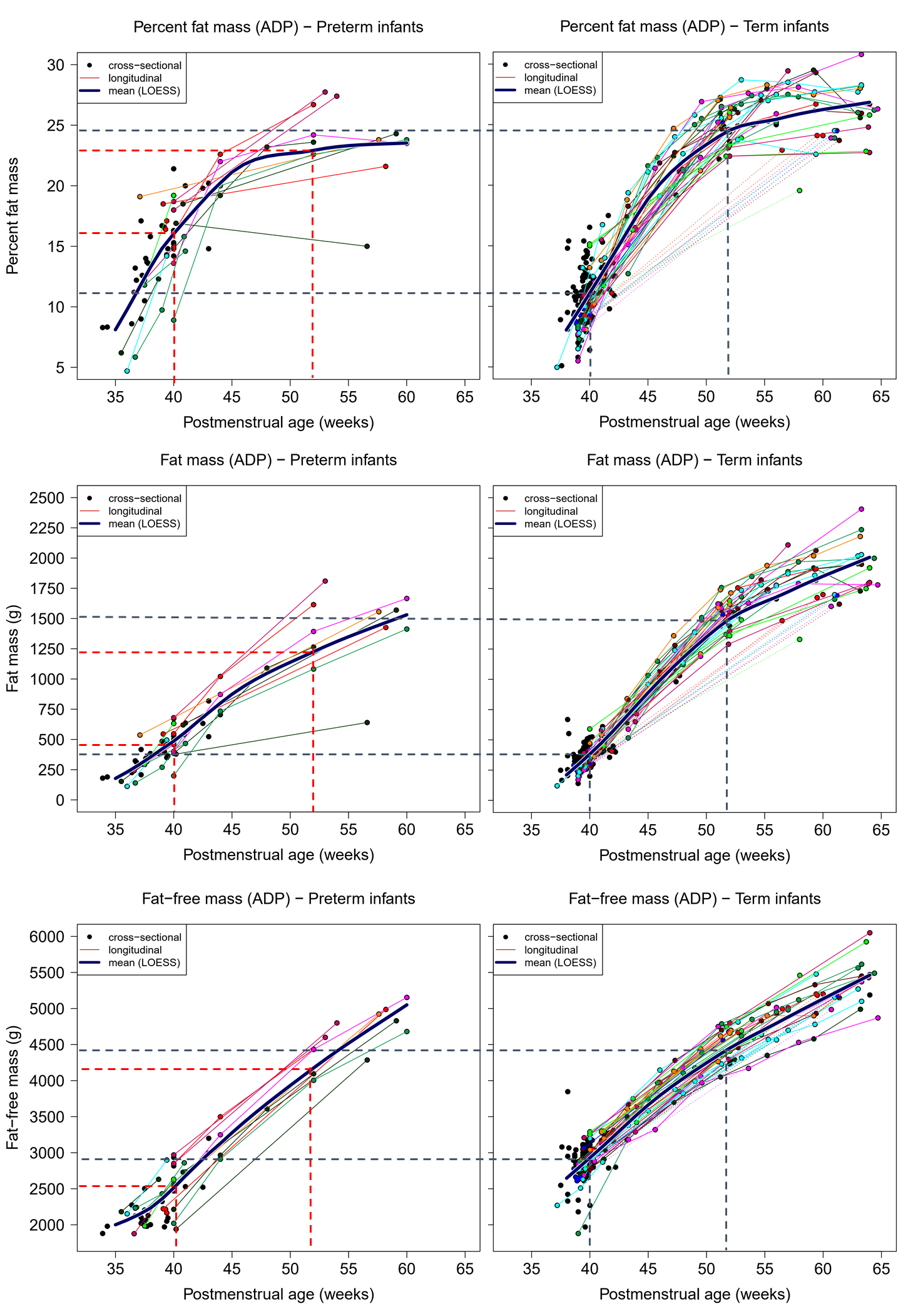


**Figure S1:** Body composition - air displacement plethysmography (APD, percent fat mass, fat mass, fat-free mass) of preterm and term infants, longitudinal data are connected by lines. Thin dotted lines show longitudinal studies which did not allow to construct trajectories because no data were available within 45 to 55 weeks, thick dotted lines indicate body composition at 40 and 52 weeks postmenstrual age (black – term infants, red – preterm infants)


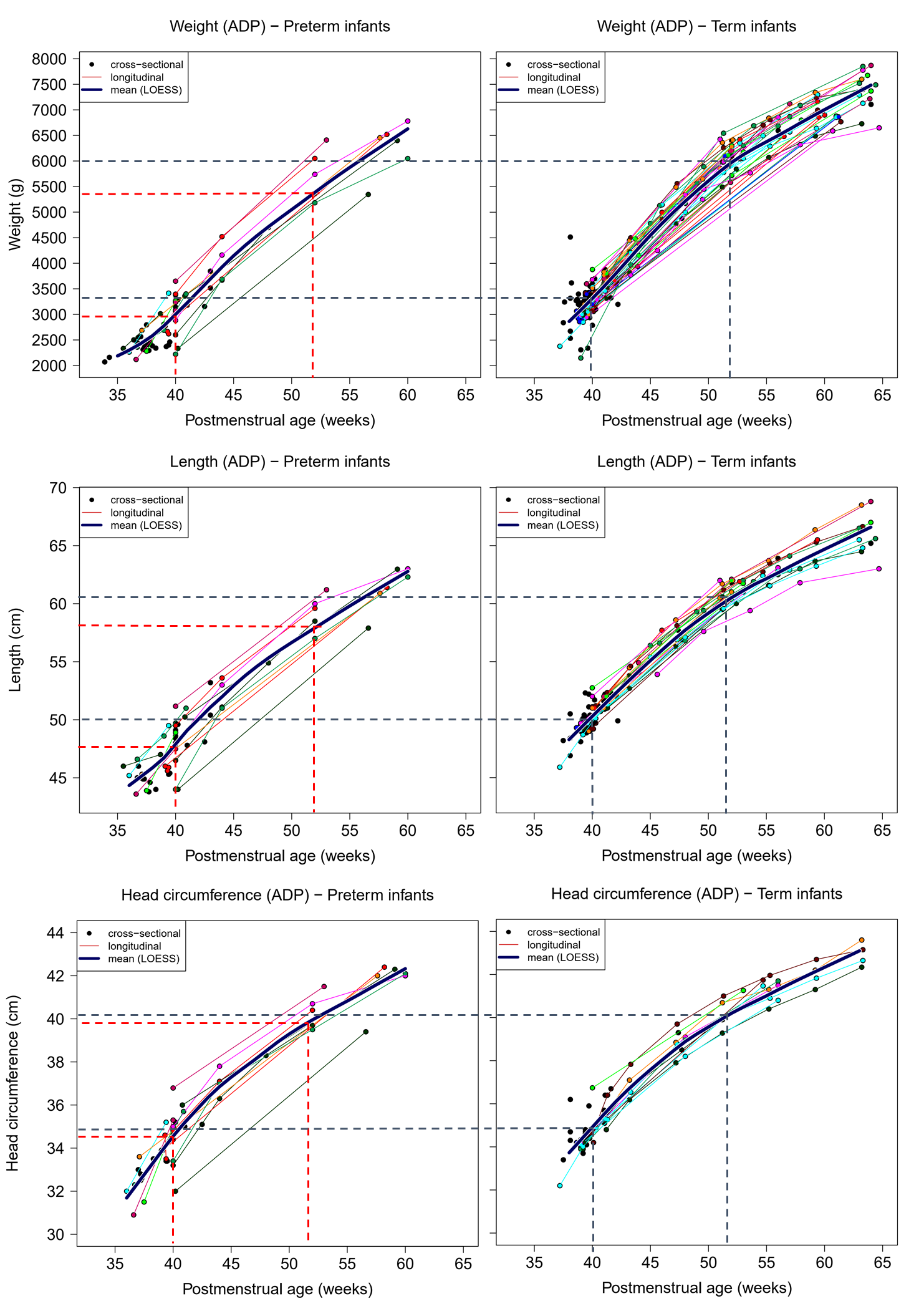


**Figure S2**: Anthropometric data (weight, length, head circumference) measured in studies using - air displacement plethysmography (APD) for postmenstrual age for preterm and term infants, thick dotted lines indicate body composition at 40 and 52 weeks postmenstrual age (black – term infants, red – preterm infants)

**
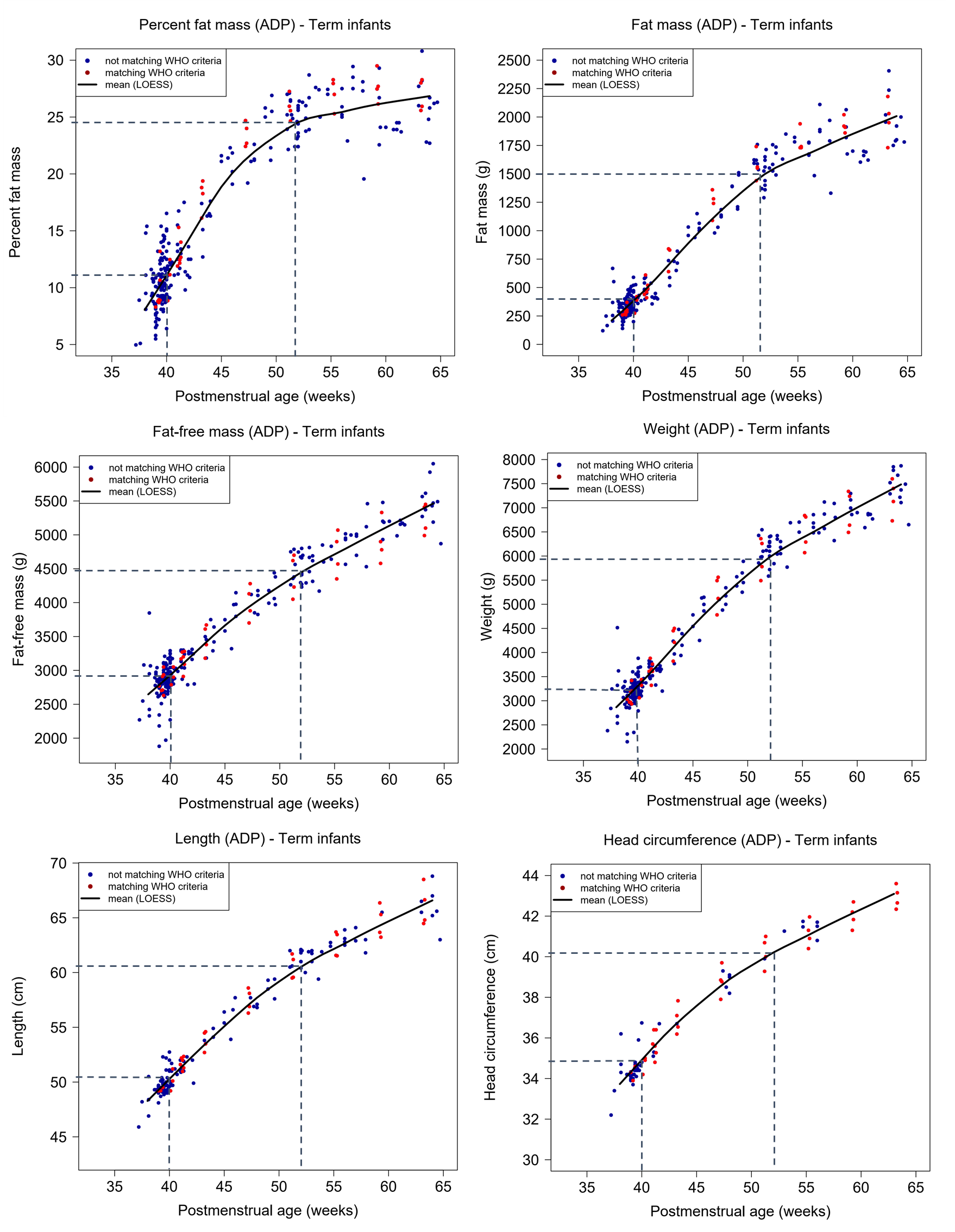
**

**Figure S3**: Comparison of body composition data (percent fat mass, fat mass, fat-free mass, weight, length, head circumference) between studies which matched the World Health Organization (WHO) Multicentre Growth Reference Study (MGRS) with other studies included in this review (blue – all studies, red – WHO MGRS), dotted lines indicate body composition at 40 and 52 weeks postmenstrual age

**Table S3**: Percent fat mass percentiles for term infants measured with ADP

| **PMA (weeks)** | **Percent fat mass percentiles of preterm infants** | | | | | | | **Percent fat mass percentiles of term infants** | | | | | | |
| --- | --- | --- | --- | --- | --- | --- | --- | --- | --- | --- | --- | --- | --- | --- |
|  | **3^rd^** | **10^th^** | **25^th^** | **50^th^** | **75^th^** | **90^th^** | **97^th^** | **3^rd^** | **10^th^** | **25^th^** | **50^th^** | **75^th^** | **90^th^** | **97^th^** |
| 35 | 3.5 | 5.1 | 6.8 | 8.6 | 10.5 | 12.1 | 13.8 |  |  |  |  |  |  |  |
| 36 | 5.1 | 6.7 | 8.4 | 10.2 | 12.0 | 13.7 | 15.3 |  |  |  |  |  |  |  |
| 37 | 6.6 | 8.2 | 9.9 | 11.7 | 13.6 | 15.2 | 16.9 |  |  |  |  |  |  |  |
| 38 | 8.1 | 9.7 | 11.4 | 13.3 | 15.1 | 16.8 | 18.4 | 4.2 | 5.5 | 6.8 | 8.2 | 9.6 | 10.9 | 12.2 |
| 39 | 9.5 | 11.2 | 12.8 | 14.7 | 16.5 | 18.2 | 19.8 | 5.7 | 6.9 | 8.2 | 9.6 | 11.0 | 12.2 | 13.4 |
| 40 | 10.9 | 12.5 | 14.2 | 16.0 | 17.9 | 19.5 | 21.2 | 7.2 | 8.4 | 9.6 | 11.0 | 12.3 | 13.6 | 14.8 |
| 41 | 12.1 | 13.7 | 15.4 | 17.3 | 19.1 | 20.8 | 22.4 | 8.8 | 10.0 | 11.2 | 12.5 | 13.8 | 15.0 | 16.2 |
| 42 | 13.2 | 14.9 | 16.6 | 18.4 | 20.3 | 21.9 | 23.6 | 10.5 | 11.7 | 12.8 | 14.1 | 15.4 | 16.6 | 17.8 |
| 43 | 14.3 | 15.9 | 17.6 | 19.5 | 21.3 | 23.0 | 24.6 | 12.3 | 13.4 | 14.6 | 15.8 | 17.1 | 18.3 | 19.4 |
| 44 | 15.2 | 16.9 | 18.6 | 20.4 | 22.3 | 24.0 | 25.6 | 14.1 | 15.2 | 16.3 | 17.6 | 18.8 | 19.9 | 21.0 |
| 45 | 16.1 | 17.8 | 19.4 | 21.3 | 23.2 | 24.8 | 26.5 | 15.7 | 16.8 | 17.9 | 19.2 | 20.4 | 21.5 | 22.6 |
| 46 | 16.9 | 18.5 | 20.2 | 22.1 | 23.9 | 25.6 | 27.3 | 17.2 | 18.3 | 19.4 | 20.6 | 21.8 | 22.9 | 24.0 |
| 47 | 17.5 | 19.2 | 20.9 | 22.7 | 24.6 | 26.3 | 27.9 | 18.4 | 19.5 | 20.6 | 21.8 | 23.0 | 24.1 | 25.2 |
| 48 | 18.1 | 19.7 | 21.4 | 23.3 | 25.1 | 26.8 | 28.5 | 19.5 | 20.6 | 21.6 | 22.8 | 24.0 | 25.1 | 26.2 |
| 49 | 18.5 | 20.1 | 21.8 | 23.7 | 25.6 | 27.3 | 28.9 | 20.3 | 21.4 | 22.5 | 23.7 | 24.9 | 26.0 | 27.0 |
| 50 | 18.8 | 20.5 | 22.1 | 24.0 | 25.9 | 27.6 | 29.2 | 21.0 | 22.1 | 23.2 | 24.4 | 25.6 | 26.7 | 27.7 |
| 51 | 19.0 | 20.6 | 22.3 | 24.2 | 26.1 | 27.8 | 29.4 | 21.6 | 22.6 | 23.7 | 24.9 | 26.1 | 27.2 | 28.3 |
| 52 | 19.1 | 20.7 | 22.4 | 24.3 | 26.2 | 27.9 | 29.5 | 22.0 | 23.1 | 24.2 | 25.4 | 26.6 | 27.7 | 28.8 |
| 53 | 19.0 | 20.7 | 22.4 | 24.2 | 26.1 | 27.8 | 29.5 | 22.4 | 23.5 | 24.6 | 25.8 | 27.0 | 28.1 | 29.2 |
| 54 | 18.8 | 20.5 | 22.2 | 24.1 | 26.0 | 27.7 | 29.3 | 22.6 | 23.7 | 24.9 | 26.1 | 27.4 | 28.5 | 29.6 |
| 55 | 18.6 | 20.3 | 22.0 | 23.9 | 25.8 | 27.5 | 29.1 | 22.8 | 23.9 | 25.1 | 26.3 | 27.6 | 28.7 | 29.9 |
| 56 | 18.4 | 20.0 | 21.7 | 23.6 | 25.5 | 27.2 | 28.9 | 22.8 | 23.9 | 25.1 | 26.4 | 27.7 | 28.9 | 30.0 |
| 57 | 18.1 | 19.8 | 21.5 | 23.4 | 25.3 | 27.0 | 28.7 | 22.7 | 23.9 | 25.1 | 26.4 | 27.7 | 28.9 | 30.1 |
| 58 | 17.9 | 19.6 | 21.3 | 23.2 | 25.1 | 26.8 | 28.4 | 22.5 | 23.7 | 24.9 | 26.3 | 27.6 | 28.8 | 30.0 |
| 59 | 17.7 | 19.4 | 21.1 | 23.0 | 24.9 | 26.6 | 28.3 | 22.3 | 23.5 | 24.8 | 26.1 | 27.5 | 28.7 | 30.0 |
| 60 | 17.5 | 19.2 | 20.9 | 22.8 | 24.7 | 26.4 | 28.1 | 22.1 | 23.3 | 24.6 | 26.0 | 27.4 | 28.7 | 29.9 |
| 61 | 17.4 | 19.0 | 20.7 | 22.7 | 24.6 | 26.3 | 27.9 | 21.9 | 23.2 | 24.5 | 25.9 | 27.3 | 28.6 | 29.9 |
| 62 | 17.2 | 18.9 | 20.6 | 22.5 | 24.4 | 26.1 | 27.8 | 21.9 | 23.2 | 24.5 | 25.9 | 27.4 | 28.7 | 30.0 |
| 63 | 17.0 | 18.7 | 20.4 | 22.3 | 24.2 | 26.0 | 27.6 | 21.9 | 23.2 | 24.5 | 26.0 | 27.4 | 28.8 | 30.1 |
| 64 | 16.9 | 18.5 | 20.3 | 22.2 | 24.1 | 25.8 | 27.5 | 21.9 | 23.2 | 24.5 | 26.0 | 27.5 | 28.9 | 30.2 |
| 65 | 16.7 | 18.4 | 20.1 | 22.0 | 23.9 | 25.6 | 27.3 | 21.9 | 23.2 | 24.6 | 26.1 | 27.6 | 28.9 | 30.3 |

**Table S4:** Fat mass percentiles for term infants measured with ADP

| **PMA (weeks)** | **Fat mass percentiles of preterm infants (gram)** | | | | | | | **Fat mass percentiles of term infants (gram)** | | | | | | |
| --- | --- | --- | --- | --- | --- | --- | --- | --- | --- | --- | --- | --- | --- | --- |
|  | **3^rd^** | **10^th^** | **25^th^** | **50^th^** | **75^th^** | **90^th^** | **97^th^** | **3^rd^** | **10^th^** | **25^th^** | **50^th^** | **75^th^** | **90^th^** | **97^th^** |
| 35 | 35 | 81 | 127 | 179 | 231 | 277 | 323 |  |  |  |  |  |  |  |
| 36 | 80 | 129 | 178 | 233 | 288 | 338 | 387 |  |  |  |  |  |  |  |
| 37 | 128 | 180 | 233 | 291 | 350 | 402 | 454 |  |  |  |  |  |  |  |
| 38 | 180 | 235 | 291 | 353 | 415 | 471 | 526 | 154 | 178 | 206 | 246 | 296 | 353 | 424 |
| 39 | 235 | 294 | 353 | 419 | 484 | 544 | 602 | 194 | 222 | 254 | 298 | 351 | 409 | 478 |
| 40 | 294 | 356 | 418 | 488 | 558 | 621 | 682 | 243 | 275 | 313 | 361 | 418 | 478 | 546 |
| 41 | 355 | 421 | 487 | 561 | 635 | 701 | 767 | 303 | 340 | 382 | 436 | 497 | 560 | 629 |
| 42 | 419 | 489 | 559 | 637 | 715 | 785 | 854 | 374 | 416 | 464 | 523 | 589 | 655 | 726 |
| 43 | 485 | 558 | 632 | 715 | 797 | 871 | 944 | 456 | 505 | 558 | 623 | 693 | 763 | 837 |
| 44 | 552 | 629 | 707 | 793 | 880 | 958 | 1035 | 549 | 603 | 662 | 733 | 808 | 881 | 958 |
| 45 | 618 | 699 | 781 | 872 | 963 | 1045 | 1125 | 650 | 710 | 774 | 850 | 931 | 1007 | 1087 |
| 46 | 683 | 768 | 853 | 949 | 1044 | 1130 | 1215 | 756 | 821 | 890 | 971 | 1056 | 1136 | 1218 |
| 47 | 746 | 834 | 924 | 1024 | 1124 | 1214 | 1302 | 862 | 932 | 1005 | 1091 | 1179 | 1262 | 1347 |
| 48 | 806 | 899 | 992 | 1097 | 1201 | 1294 | 1387 | 964 | 1039 | 1117 | 1206 | 1297 | 1382 | 1469 |
| 49 | 864 | 960 | 1057 | 1165 | 1274 | 1371 | 1467 | 1061 | 1140 | 1221 | 1313 | 1407 | 1494 | 1582 |
| 50 | 917 | 1017 | 1118 | 1230 | 1342 | 1444 | 1543 | 1150 | 1231 | 1315 | 1410 | 1507 | 1595 | 1684 |
| 51 | 966 | 1070 | 1174 | 1290 | 1406 | 1511 | 1614 | 1229 | 1313 | 1399 | 1496 | 1595 | 1684 | 1773 |
| 52 | 1011 | 1117 | 1225 | 1345 | 1464 | 1572 | 1678 | 1296 | 1383 | 1471 | 1571 | 1670 | 1761 | 1851 |
| 53 | 1051 | 1160 | 1270 | 1393 | 1516 | 1627 | 1736 | 1354 | 1443 | 1534 | 1635 | 1736 | 1828 | 1918 |
| 54 | 1086 | 1197 | 1310 | 1436 | 1561 | 1675 | 1786 | 1402 | 1494 | 1586 | 1689 | 1792 | 1885 | 1976 |
| 55 | 1116 | 1230 | 1345 | 1473 | 1601 | 1717 | 1830 | 1440 | 1534 | 1629 | 1734 | 1839 | 1932 | 2025 |
| 56 | 1142 | 1258 | 1376 | 1506 | 1636 | 1754 | 1870 | 1469 | 1566 | 1663 | 1770 | 1876 | 1971 | 2064 |
| 57 | 1166 | 1284 | 1403 | 1535 | 1668 | 1787 | 1905 | 1490 | 1589 | 1688 | 1797 | 1905 | 2001 | 2095 |
| 58 | 1188 | 1307 | 1428 | 1562 | 1696 | 1817 | 1937 | 1505 | 1606 | 1708 | 1819 | 1928 | 2026 | 2121 |
| 59 | 1208 | 1329 | 1451 | 1587 | 1723 | 1846 | 1967 | 1516 | 1620 | 1723 | 1837 | 1948 | 2047 | 2143 |
| 60 | 1227 | 1349 | 1473 | 1611 | 1749 | 1873 | 1996 | 1525 | 1632 | 1738 | 1853 | 1967 | 2067 | 2164 |
| 61 | 1246 | 1370 | 1496 | 1635 | 1775 | 1900 | 2024 | 1535 | 1645 | 1754 | 1872 | 1988 | 2090 | 2188 |
| 62 | 1265 | 1391 | 1518 | 1659 | 1800 | 1927 | 2053 | 1548 | 1661 | 1774 | 1895 | 2013 | 2117 | 2217 |
| 63 | 1284 | 1411 | 1540 | 1683 | 1826 | 1955 | 2082 | 1561 | 1679 | 1794 | 1919 | 2040 | 2146 | 2248 |
| 64 | 1303 | 1432 | 1562 | 1707 | 1852 | 1982 | 2110 | 1574 | 1696 | 1815 | 1942 | 2066 | 2174 | 2278 |
| 65 | 1322 | 1452 | 1584 | 1731 | 1877 | 2009 | 2139 | 1586 | 1712 | 1834 | 1965 | 2091 | 2201 | 2306 |

**Table S5:** Fat-free mass percentiles for term infants measured with ADP

| **PMA (weeks)** | **Fat-free mass percentiles of preterm infants (gram)** | | | | | | | **Fat-free mass percentiles of term infants (gram)** | | | | | | |
| --- | --- | --- | --- | --- | --- | --- | --- | --- | --- | --- | --- | --- | --- | --- |
|  | **3^rd^** | **10^th^** | **25^th^** | **50^th^** | **75^th^** | **90^th^** | **97^th^** | **3^rd^** | **10^th^** | **25^th^** | **50^th^** | **75^th^** | **90^th^** | **97^th^** |
| 35 | 1763 | 1831 | 1901 | 1979 | 2056 | 2126 | 2195 |  |  |  |  |  |  |  |
| 36 | 1798 | 1884 | 1970 | 2067 | 2163 | 2250 | 2335 |  |  |  |  |  |  |  |
| 37 | 1832 | 1937 | 2043 | 2161 | 2279 | 2386 | 2491 |  |  |  |  |  |  |  |
| 38 | 1871 | 1996 | 2123 | 2265 | 2406 | 2534 | 2659 | 2191 | 2334 | 2479 | 2641 | 2802 | 2947 | 3090 |
| 39 | 1923 | 2068 | 2216 | 2379 | 2543 | 2691 | 2836 | 2355 | 2491 | 2628 | 2781 | 2934 | 3071 | 3207 |
| 40 | 1999 | 2160 | 2324 | 2506 | 2688 | 2852 | 3013 | 2519 | 2648 | 2779 | 2925 | 3070 | 3201 | 3330 |
| 41 | 2101 | 2273 | 2448 | 2642 | 2837 | 3012 | 3184 | 2682 | 2806 | 2932 | 3071 | 3211 | 3337 | 3461 |
| 42 | 2228 | 2406 | 2586 | 2786 | 2987 | 3167 | 3345 | 2843 | 2963 | 3085 | 3220 | 3355 | 3477 | 3597 |
| 43 | 2375 | 2553 | 2734 | 2935 | 3136 | 3316 | 3495 | 3000 | 3117 | 3237 | 3369 | 3501 | 3620 | 3738 |
| 44 | 2536 | 2711 | 2889 | 3086 | 3284 | 3462 | 3637 | 3152 | 3268 | 3386 | 3516 | 3647 | 3764 | 3880 |
| 45 | 2702 | 2873 | 3046 | 3239 | 3431 | 3604 | 3775 | 3299 | 3414 | 3530 | 3660 | 3790 | 3906 | 4021 |
| 46 | 2870 | 3036 | 3204 | 3390 | 3577 | 3745 | 3911 | 3438 | 3553 | 3670 | 3799 | 3929 | 4045 | 4160 |
| 47 | 3037 | 3197 | 3360 | 3540 | 3721 | 3884 | 4044 | 3569 | 3685 | 3802 | 3932 | 4062 | 4179 | 4295 |
| 48 | 3199 | 3355 | 3513 | 3688 | 3863 | 4021 | 4177 | 3693 | 3809 | 3927 | 4058 | 4190 | 4308 | 4424 |
| 49 | 3354 | 3506 | 3660 | 3832 | 4003 | 4157 | 4309 | 3808 | 3926 | 4045 | 4178 | 4311 | 4431 | 4549 |
| 50 | 3501 | 3651 | 3802 | 3970 | 4138 | 4290 | 4439 | 3915 | 4035 | 4157 | 4292 | 4427 | 4548 | 4668 |
| 51 | 3639 | 3787 | 3937 | 4103 | 4269 | 4419 | 4566 | 4014 | 4137 | 4260 | 4398 | 4535 | 4659 | 4781 |
| 52 | 3768 | 3914 | 4063 | 4228 | 4393 | 4542 | 4689 | 4105 | 4230 | 4356 | 4497 | 4637 | 4763 | 4888 |
| 53 | 3886 | 4032 | 4181 | 4345 | 4510 | 4658 | 4804 | 4188 | 4316 | 4445 | 4588 | 4732 | 4861 | 4988 |
| 54 | 3996 | 4142 | 4290 | 4454 | 4619 | 4767 | 4913 | 4265 | 4396 | 4528 | 4675 | 4821 | 4953 | 5084 |
| 55 | 4100 | 4245 | 4392 | 4556 | 4720 | 4867 | 5012 | 4337 | 4471 | 4606 | 4757 | 4907 | 5043 | 5176 |
| 56 | 4199 | 4343 | 4489 | 4651 | 4813 | 4959 | 5103 | 4406 | 4543 | 4682 | 4836 | 4990 | 5129 | 5266 |
| 57 | 4295 | 4437 | 4581 | 4741 | 4901 | 5045 | 5188 | 4471 | 4612 | 4754 | 4913 | 5071 | 5214 | 5355 |
| 58 | 4389 | 4529 | 4671 | 4828 | 4985 | 5127 | 5267 | 4533 | 4678 | 4825 | 4988 | 5151 | 5297 | 5442 |
| 59 | 4482 | 4619 | 4758 | 4912 | 5066 | 5205 | 5342 | 4594 | 4743 | 4894 | 5061 | 5229 | 5380 | 5529 |
| 60 | 4573 | 4708 | 4844 | 4995 | 5146 | 5282 | 5416 | 4653 | 4806 | 4962 | 5134 | 5307 | 5462 | 5616 |
| 61 | 4665 | 4796 | 4929 | 5077 | 5225 | 5358 | 5490 | 4711 | 4869 | 5030 | 5208 | 5385 | 5546 | 5704 |
| 62 | 4756 | 4885 | 5015 | 5160 | 5304 | 5435 | 5563 | 4769 | 4932 | 5097 | 5281 | 5465 | 5630 | 5793 |
| 63 | 4847 | 4973 | 5101 | 5242 | 5384 | 5511 | 5637 | 4825 | 4993 | 5164 | 5354 | 5544 | 5715 | 5884 |
| 64 | 4939 | 5062 | 5186 | 5325 | 5463 | 5587 | 5710 | 4879 | 5054 | 5231 | 5427 | 5624 | 5801 | 5975 |
| 65 | 5030 | 5150 | 5272 | 5407 | 5542 | 5664 | 5784 | 4933 | 5113 | 5297 | 5500 | 5704 | 5887 | 6067 |


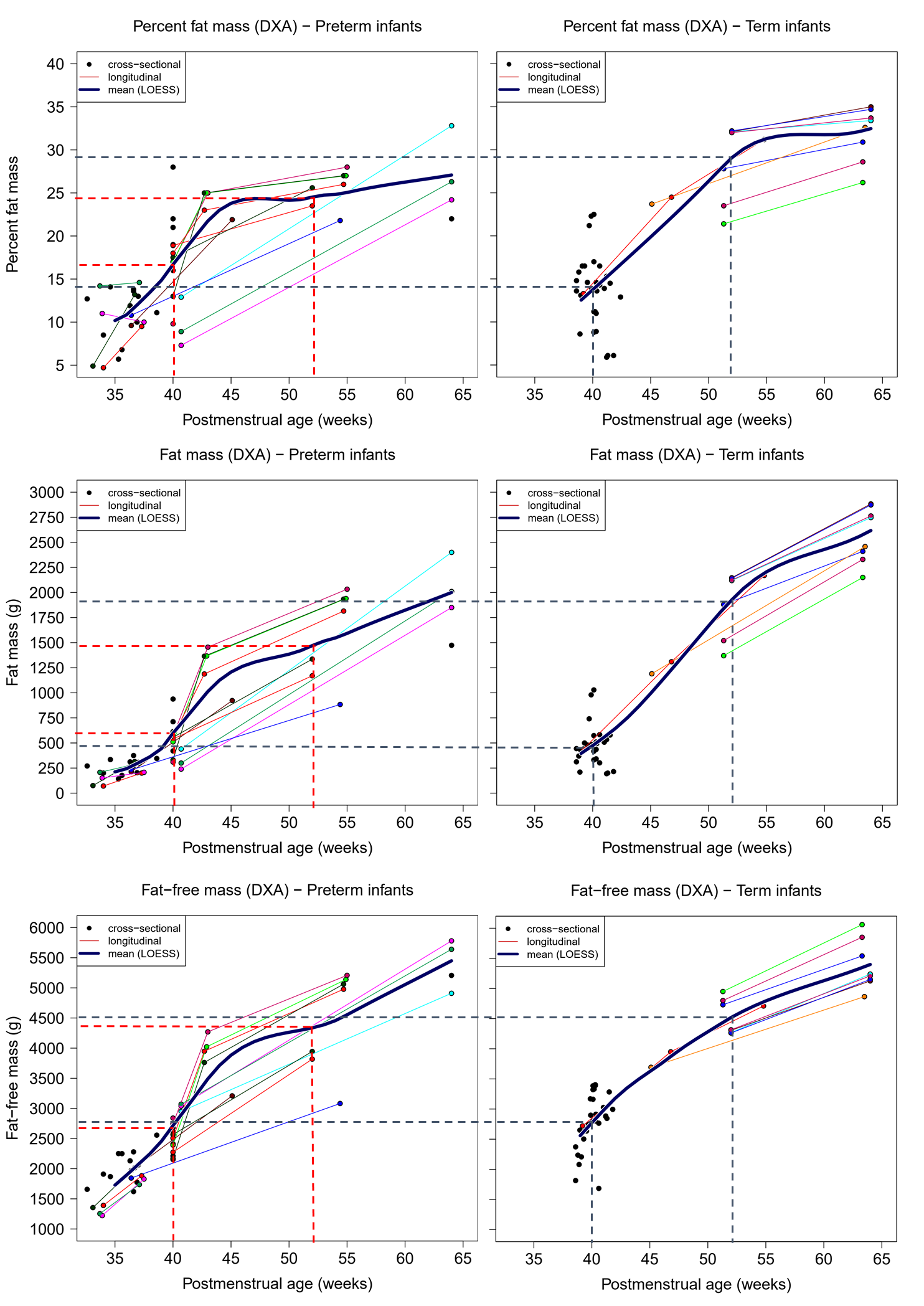


**Figure S4:** Body composition (percent fat mass, fat mass, fat-free mass) for postmenstrual age in preterm and term infants measured with dual energy x-ray absorptiometry (DXA), dotted lines indicate body composition at 40 and 52 weeks postmenstrual age (black – term infants, red – preterm infants)


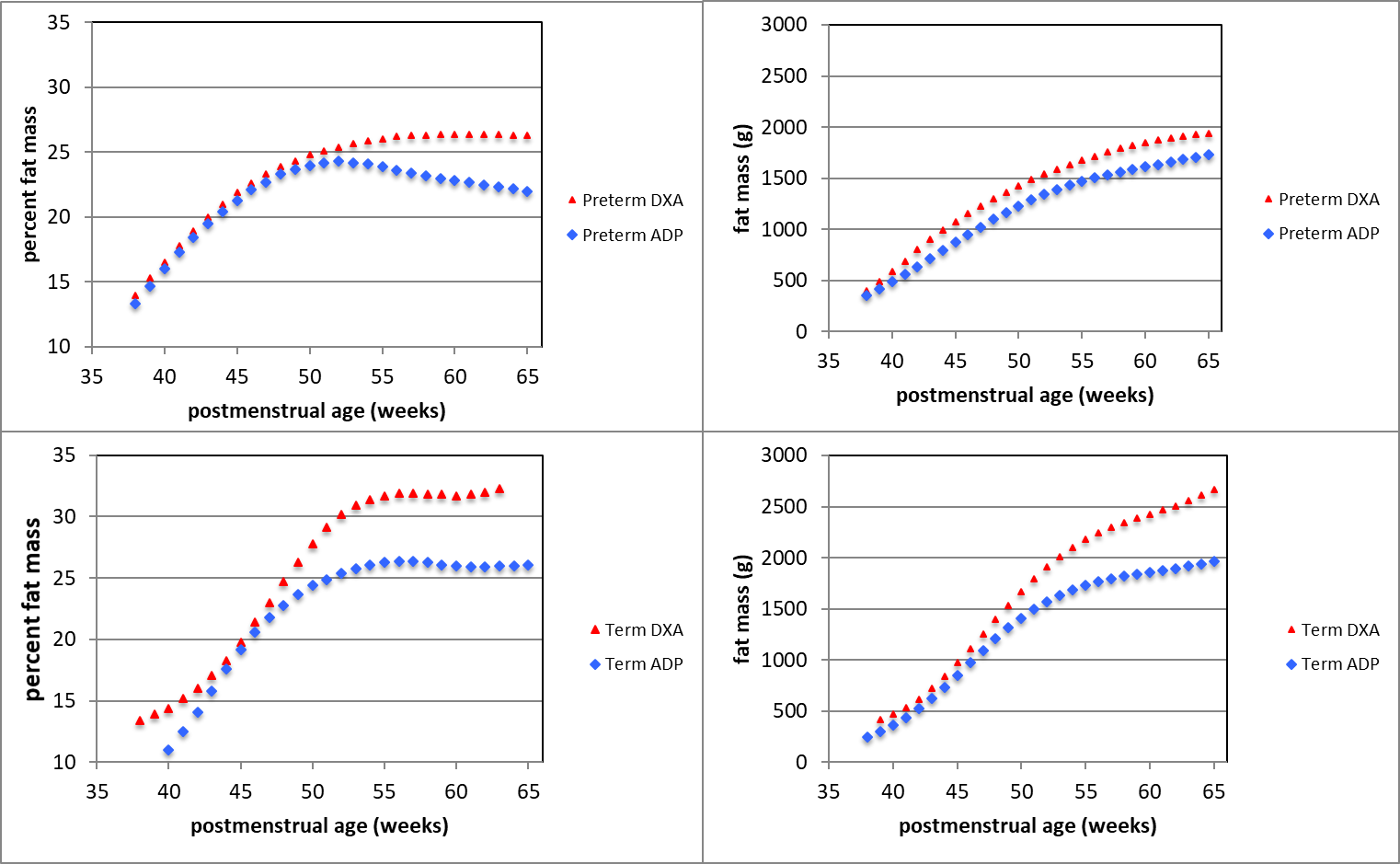


**Figure S5:** Comparison of ADP and DXA measurements in preterm and term infants for postmenstrual age. Dotted lines present median data of body composition.
